# MAP4K1 and MAP4K2 regulate ABA-induced and Ca^2+^-mediated stomatal closure in *Arabidopsis*

**DOI:** 10.1126/sciadv.adt4916

**Published:** 2025-12-19

**Authors:** Kota Yamashita, Sotaro Katagiri, Hinano Takase, Yangdan Li, Anzu Oishi, Airi Otoguro, Yoshiaki Kamiyama, Shota Yamauchi, Yuh-Shuh Wang, Atsushi Takemiya, Izumi C. Mori, Hannes Kollist, Taishi Umezawa

**Affiliations:** ^1^ Graduate School of Bio-Applications and Systems Engineering, Tokyo University of Agriculture and Technology, Koganei 184-8588, Tokyo, Japan.; ^2^ Graduate School of Advanced Interdisciplinary Science, Tokyo University of Agriculture and Technology, Koganei 184-8588, Tokyo, Japan.; ^3^ Department of Biology, Graduate School of Sciences and Technology for Innovation, Yamaguchi University, Yamaguchi 753-8512, Japan.; ^4^ Faculty of Science and Technology, University of Tartu, Tartu 50090, Estonia.; ^5^ Institute of Plant Science and Resources, Okayama University, Kurashiki 710-0046, Okayama, Japan.; ^6^ Faculty of Agriculture, Tokyo University of Agriculture and Technology, Fuchu 183-8538, Tokyo, Japan.

## Abstract

Abscisic acid (ABA)–induced stomatal closure limits water loss from plants under drought stress. To investigate the signaling pathways involved in ABA-induced stomatal closure, we performed a phosphoproteomic analysis of ABA-treated *Arabidopsis* guard cell protoplasts (GCPs). We found that ABA-responsive phosphorylation of MITOGEN-ACTIVATED PROTEIN 4 KINASE 1 (MAP4K1) was significantly down-regulated in SnRK2-disrupted mutants. Subsequent biochemical assays showed that Ser^479^ of MAP4K1 is directly phosphorylated by SRK2E/OST1, a central ABA kinase. Mutational analyses of MAP4K1 and MAP4K2 revealed that both kinases positively regulate ABA-induced stomatal closure and that Ser^479^ of MAP4K1 is required for this phenotype. In *map4k1map4k2* double mutants, stomatal closure was induced by applying exogenous Ca^2+^ but not H_2_O_2_. Electrophysiological experiments showed that MAP4K1/2 is required for ABA-dependent activation of Ca^2+^-permeable channels in GCPs. Together, our results indicate that SnRK2 and MAP4K function as a signaling module to regulate the Ca^2+^-mediated pathway in ABA-induced stomatal closure.

## Teaser

MAP4K1/2 is directly phosphorylated by SRK2E/OST1 to regulate ABA-induced and Ca^2+^-mediated stomatal closure in *Arabidopsis*.

## INTRODUCTION

Stomata are small pores on the leaf epidermis that mediate gas exchange with the surrounding atmosphere. Each stoma consists of a pair of guard cells surrounding a central pore. Stomatal aperture is tightly controlled in response to environmental changes. Stomata open in response to light and low CO_2_ concentration to promote gas exchange and transpiration for photosynthetic carbon assimilation and maintenance of leaf temperature. Conversely, stomata close to prevent water loss when plants experience drought or osmotic stress. The regulatory pathway of stomatal movements is crucial for plant growth and stress responses.

Stomatal closure is largely dependent on the phytohormone abscisic acid (ABA), and subclass III SNF1-related protein kinase 2 (SnRK2) kinases play a crucial role in ABA signaling (*1*, *2*). SRK2E/OPEN STOMATA 1 (OST1) is a member of subclass III SnRK2 and plays a primary role in ABA-induced stomatal closure (*3*–*5*). ABA induces the activation of SRK2E/OST1, which directly phosphorylates SLOW ANION CHANNEL–ASSOCIATED 1 (SLAC1), thereby promoting anion efflux (*3*–*5*). On the other hand, ABA induces an increase in reactive oxygen species (ROS) and cytoplasmic Ca^2+^ concentration ([Ca^2+^]_cyt_) in guard cells (*3*–*5*). In addition to activation of SRK2E/OST1, ABA-dependent [Ca^2+^]_cyt_ elevation activates several Ca^2+^-dependent protein kinases (CDPKs) and a pair of calcineurin-B–like proteins (CBLs) and CBL-interacting protein kinases (CIPKs) to phosphorylate SLAC1 or SLAH3 (SLAC1 homolog 3) (*4*, *5*). These processes result in plasma membrane depolarization and a reduction in guard cell volume, leading to stomatal closure (*3*). Therefore, both Ca^2+^-dependent and Ca^2+^-independent pathways coordinate ABA-induced stomatal closure (*3*–*5*).

ROS induce the activation of plasma membrane Ca^2+^-permeable (*I*_Ca_) channels (*6*–*8*). SRK2E/OST1 is involved in this process on the basis of several lines of evidence. ABA-dependent ROS production and [Ca^2+^]_cyt_ elevation are nearly abolished in a *srk2e/ost1* mutant, and SRK2E/OST1 phosphorylates the membrane-bound and ROS-producing NADPH (reduced form of nicotinamide adenine dinucleotide phosphate) oxidases RBOHF and RBOHFD (RESPIRATORY BURST OXIDASE HOMOLOG F and D, respectively) to promote ROS production (*9*–*13*). These findings suggest a connection between SRK2E/OST1 and the Ca^2+^-dependent pathway, although a mechanistic explanation for this connection remains unknown.

In this study, we identified MITOGEN-ACTIVATED PROTEIN 4 KINASE 1 (MAP4K1/AtMAP4Kα1) and MAP4K2 as positive regulators of ABA-induced stomatal closure. Our results further demonstrate that SRK2E/OST1 directly phosphorylates MAP4K1 at Ser^479^ and that MAP4K1/2 mediates ABA signaling to elevate [Ca^2+^]_cyt_. Together, our results substantially expand current models of ABA-induced stomatal closure.

## RESULTS

### Overview of phosphoproteomic analysis using *Arabidopsis* guard cell protoplasts

**Fig. 1.**
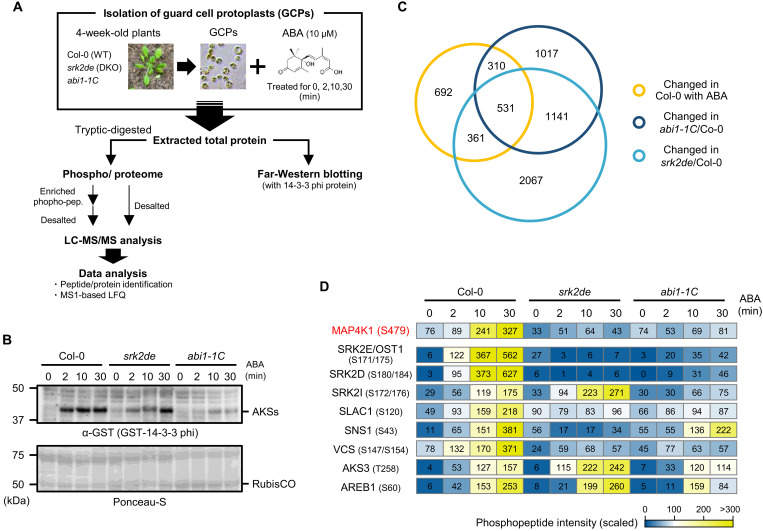
Overview of phosphoproteomic analysis using *Arabidopsis* guard cells. (**A**) Workflow of the phosphoproteomic analysis for *Arabidopsis* GCPs. Protein extracts of GCPs were prepared from Col-0 as wild-type (WT), *srk2de* double-knockout mutant (DKO), and *abi1-1C* treated with 10 μM ABA for indicated period. The total protein samples were supplied for LC-MS/MS analysis or Western blotting. (**B**) Far-Western blotting analysis of 14-3-3 protein. GST-tagged 14-3-3 (GF14phi) protein was used as a probe. Black arrows and open arrows indicate the position of AKSs and RubisCO, respectively. This experiment was repeated four times biologically independent with similar results. (**C**) The Venn diagram shows the overlap between ABA-responsive phosphopeptides in Col-0 and up/down-regulated phosphopeptides in *srk2de* or *abi1-1C*. (**D**) Heatmap showing the intensity of phosphopeptides from known or putative SnRK2 substrates. To compare the peptide abundance between samples under different conditions, the mean intensities were scaled to the same range for each phosphopeptide.

Previous phosphoproteomic studies of ABA-induced responses in *Arabidopsis* seedlings have identified multiple SnRK2 substrates (*14*–*16*). In this study, we carried out a cell type–specific phosphoproteomic analysis to identify SnRK2 substrates in guard cells (Fig. 1A). Using a previously developed protocol (*17*, *18*), guard cell protoplasts (GCPs) were isolated from leaves of *Arabidopsis* wild type (Col-0) and two types of ABA-insensitive mutants: *srk2de*, a double knockout mutant of SRK2D/SnRK2.2 and SRK2E/OST1, and *abi1-1C*, a gain-of-function mutant of type 2C protein phosphatase ABI1 (ABA INSENSITIVE 1) (*19*). Before phosphoproteomic analysis, a far-Western blot analysis was performed to confirm ABA-induced phosphorylation of ABA-RESPONSIVE KINASE SUBSTRATE (AKS) transcription factors (*20*, *21*). As reported previously (*20*, *21*), AKSs were phosphorylated in Col-0 GCPs, and levels of AKS phosphorylation were reduced in *srk2de* and *abi1-1C* GCPs (Fig. 1B). These results confirm that our enriched GCPs are ABA-responsive.

For liquid chromatography–tandem mass spectrometry (LC-MS/MS) analysis, total protein samples were extracted from Col-0, *srk2de*, and *abi1-1C* GCPs and then digested with trypsin. Subsequently, the digested peptides were divided into two parts: enrichment of phosphorylation for phosphoproteomics or not for conventional proteomics (Fig. 1A). To support the phosphoproteomics, the proteomic samples were prepared. From all LC-MS/MS runs, we identified a total of 10,356 protein groups, which overlapped with 94.5% of protein groups previously identified in *Arabidopsis* GCPs (fig. S1A and table S1) (*22*). In the proteomic data, 8451 unique protein groups were detected, and 7762 proteins were reproducibly quantified in any sample group (table S2). Among them, 374 proteins were significantly up-regulated or down-regulated after ABA treatment in Col-0 (table S2). On the other hand, 6177 unique proteins were detected in the phosphoproteomics (table S1). Comparing proteins identified in the former with the latter, 1905 (30.8%) of protein groups were only detected in the latter (fig. S1B).

A total of 24,887 peptide groups identified in the phosphoproteomics contained 17,529 (70.4%) of unique phosphorylation sites, and 14,645 (58.8%) of the phosphopeptides were reproducibly quantified (fig. S1C and table S3). Among them, 1894 phosphopeptides were significantly up-regulated or down-regulated after ABA treatment in Col-0 (Fig. 1C and table S3). Furthermore, of these ABA-responsive peptides, 1202 were also significantly changed in *srk2de* or *abi1-1C* compared to Col-0 (Fig. 1C and table S3). Gene Ontology (GO) analysis of proteins associated with significantly altered phosphopeptides revealed an enrichment of several GO terms, including “stomatal movement (GO: 0010119),” “chromatin remodeling (GO: 0006338),” and “cytoskeletal organization (GO: 0007010)” (fig. S1D and table S4).

Several phosphopeptides derived from the activation loop of subclass III SnRK2s accumulated to significantly higher levels in response to ABA (Fig. 1D). However, consistent with Fig. 1B, these same phosphopeptides did not increase in abundance in GCP samples from *srk2de* or *abi1-1C* (Fig. 1D). In addition to these SnRK2-derived phosphopeptides, phosphopeptides from several known SnRK2 substrate proteins, including SLAC1, AKS3/FBH4, ABF1/AREB2, SNS1 (SnRK2-Substrate 1), and VCS (VARICOSE) (*14*), differentially accumulated in ABA-treated GCPs isolated from Col-0, suggesting that our data broadly captured ABA-induced phosphorylation events in guard cells and therefore could be useful for identifying previously unknown SnRK2 substrates (Fig. 1D).

### SRK2E/OST1 directly phosphorylates MAP4K1 at Ser^479^

**Fig. 2.**
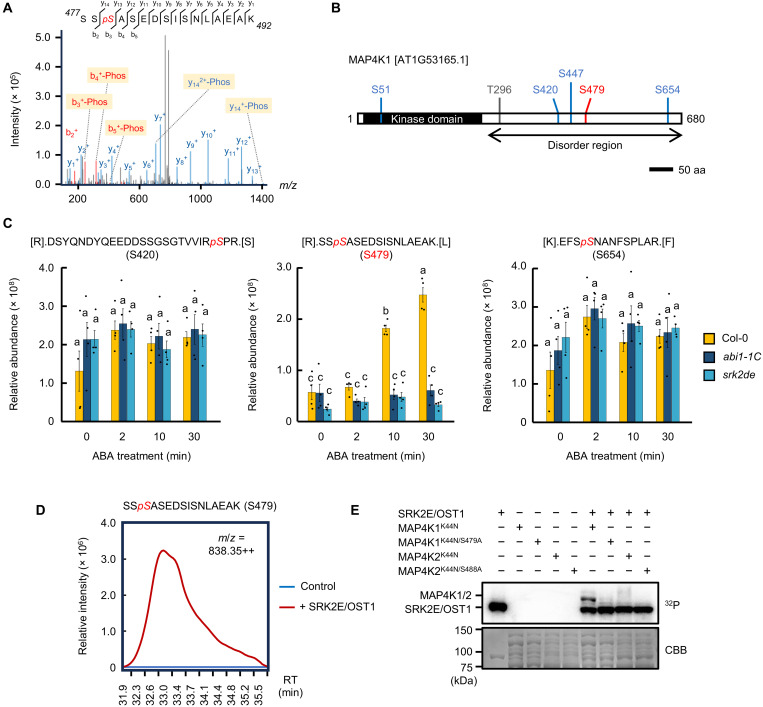
SRK2E/OST1 phosphorylates MAP4K1 at Ser^479^ in vivo. (**A**) MS/MS spectra derived from the in vivo phosphopeptide containing Ser^479^ of MAP4K1 are shown (Theo. MH+ = 1675.70583 Da; Obser. MH+ = 1675.70881 Da). (**B**) Domain structure of *Arabidopsis* MAP4K1. Phosphorylation sites detected in this study are depicted as red or blue lines. aa, amino acids. (**C**) Relative abundance of MAP4K1 peptides containing phosphorylated Ser^420^, Ser^479^, or Ser^654^. Data represent the means ± SE (*n* = 4, biologically independent replicate). Different letters indicate significant differences (Tukey’s test, *P* < 0.05). (**D**) In vitro phosphorylation assay was conducted using a kinase-dead form of MBP-MAP4K1 (MAP4K1^K44N^) with or without MBP-tagged SRK2E/OST1, followed by LC-MS/MS analysis. The extracted ion chromatogram of a MAP4K1 peptide [SS*p*SASEDSISNLAEAK] containing phosphorylated Ser^479^ in the presence or absence of SRK2E. RT, retention time. (**E**) In vitro phosphorylation assay for SRK2E and MAP4K1/2. MBP-SRK2E was incubated with MBP-MAP4K1/2^K44N^, MBP-MAP4K1^K44N/S479A^, or MBP-MAP4K2^K44N/S488A^ in the presence of [γ-^32^P]ATP. Phosphorylation levels were detected by autoradiography. CBB (Coomassie brilliant blue) staining showed protein loading in each lane.

Among the phosphopeptides identified in ABA-treated GCPs, a phosphopeptide from MAP4K1/AtMAP4Kα1 exhibited a similar trend to known SnRK2 substrates, suggesting that MAP4K1 may be an SnRK2 substrate (Fig. 1D). MAP4K1 is a member of the MAP4K gene family (fig. S2), and MAP4K2 is ~80% identical to MAP4K1 (*23*). MS/MS spectra clearly show the phosphorylation of Ser^479^ at MAP4K1 in *Arabidopsis* guard cells (Fig. 2A and fig. S3). In addition, our dataset contained six phosphorylation sites in MAP4K1 (Fig. 2B) and three phosphorylation sites in MAP4K2 (fig. S4, A and B). Ser^479^ of MAP4K1 was significantly up-regulated in response to ABA in Col-0 but not in *srk2de* and *abi1-1C* (Fig. 2C). Other phosphorylation sites, such as Ser^420^ or Ser^654^, remained unchanged (Fig. 2C). Ser^488^ of MAP4K2 is located in a similar position relative to Ser^479^ of MAP4K1, but a corresponding phosphopeptide was not detected in this study (fig. S4A).

To investigate whether MAP4K1 and MAP4K2 are direct substrates of SRK2E/OST1, an in vitro phosphorylation assay was performed by incubating *Escherichia coli*–expressed maltose-binding protein (MBP)–tagged SRK2E/OST1 together with MAP4K1^K44N^ or MAP4K2^K44N^ in a kinase reaction buffer. After incubation, the proteins were analyzed by LC-MS/MS, revealing that MBP-SRK2E phosphorylated MAP4K1 at Ser^479^ (Fig. 2D). In a separate in vitro phosphorylation assay, MBP-MAP4K1^K44N^ was phosphorylated by MBP-SRK2E, but MAP4K1^K44N/S479A^ was not (Fig. 2E), confirming that SRK2E phosphorylates Ser^479^ of MAP4K1. On the other hand, the level of SRK2E phosphorylation of MBP-MAP4K2^K44N^ was lower than that of MBP-MAP4K1^K44N^ (Fig. 2E).

### IP-MS analysis of MAP4K1-interacting proteins

**Fig. 3.**
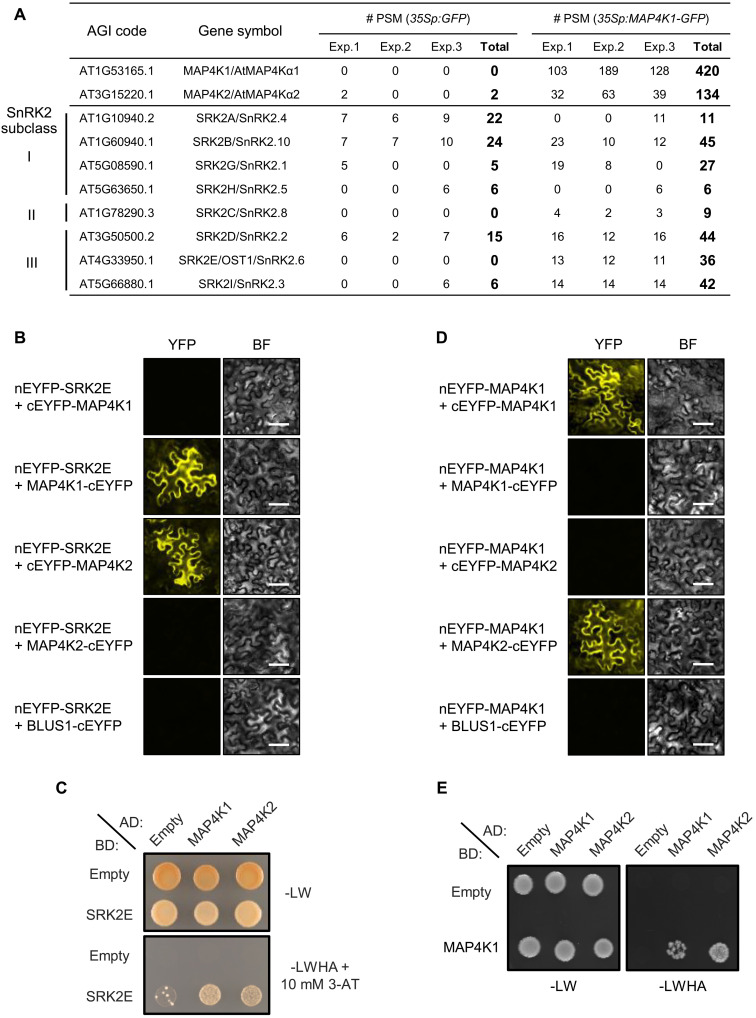
MAP4K1 interacts with MAP4K2 and subclass III SnRK2s. (**A**) IP-MS analysis of *35Sp:GFP* and *35Sp:MAP4K1-GFP* transgenic plants. Immunoprecipitation was performed using an anti-GFP antibody, followed by LC-MS/MS analysis. The peptide spectrum match (PSM) score indicates the number of each peptide detected in this experiment. AGI, Arabidopsis Genome Initiative. (**B**) BiFC assays were conducted for SRK2E/OST1 and MAP4K1/2 in *N. benthamiana* epidermal cells. nEYFP and cEYFP represent the N- and C-terminal fragments of the enhanced yellow fluorescent protein (EYFP), respectively. BLUS1/MAP4K10 was used as the negative control. BF indicates bright-field images. Scale bars indicate 50 μm. (**C**) Y2H assay between SRK2E/OST1 and MAP4K1. Yeast cells were grown on nonselective SD-Leu/-Trp (-LW) or selective SD-Leu/-Trp/-His/-Ade (-LWHA) with 10 mM 3-AT (3-amino-1,2,4-triazole) media at 30°C for 7 days. (**D**) BiFC assays between MAP4K1 and MAP4K2 in *N. benthamiana* epidermal cells. Scale bars indicate 50 μm. (**E**) Y2H assay between MAP4K1 and MAP4K2. Yeast cells were grown on nonselective SD-Leu/-Trp (-LW) or selective SD-Leu/-Trp/-His/-Ade (-LWHA) media at 30°C for 7 days.

To identify MAP4K1-interacting proteins, we next did immunoprecipitation–mass spectrometry (IP-MS) analysis using *35Sp:MAP4K1-GFP* or *35Sp:GFP* transgenic lines. In this analysis, several SnRK2s were detected, suggesting that MAP4K1 preferentially interacts with subclass III SnRK2s (SRK2D/E/I) (Fig. 3A and table S4). Bimolecular fluorescence complementation (BiFC) assays confirmed that SRK2E/OST1 interacts with MAP4K1 and MAP4K2 in the cytosol of *Nicotiana benthamiana* epidermal cells but not with BLUE LIGHT SIGNALING 1 (BLUS1), also known as MAP4K10 (Fig. 3B). These data are consistent with the subcellular localization of MAP4K1-green fluorescent protein (GFP) and SRK2E-GFP in the cytosol of *Arabidopsis* mesophyll cell protoplasts (MCPs) (fig. S5). In addition, the interaction between SRK2E and MAP4K1/2 was further supported by yeast two-hybrid (Y2H) assay (Fig. 3C) and in vitro pull-down assay using MBP-MAP4K1/2 and glutathione *S*-transferase (GST)–tagged SRK2E (fig. S6).

In addition to SnRK2s-MAP4K1 interactions, the IP-MS analysis also detected an interaction between MAP4K1 and MAP4K2 (Fig. 3A and table S5). A BiFC assay and Y2H assay confirmed that not only do MAP4K1 and MAP4K2 directly interact as a heterodimer but also that MAP4K1 forms a homodimer (Fig. 3, D and E). Together, our results reveal that MAP4K1 interacts with subclass III SnRK2s and MAP4K1/2 in planta.

### MAP4K1 and MAP4K2 redundantly function in ABA-induced stomatal closure

**Fig. 4.**
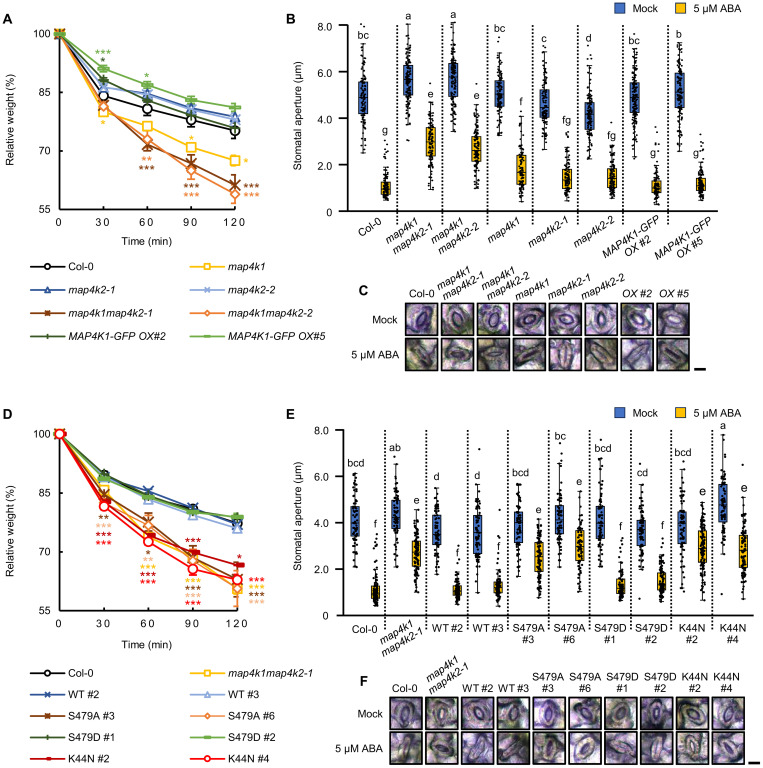
MAP4K1 and MAP4K2 positively regulate ABA-induced stomatal closure. (**A**) Water loss from detached leaves of Col-0–, *map4k1*-, *map4k2-1/2–*, and *MAP4K1-GFP*–overexpressing plants. Data are the means ± SE (*n* = 3), and asterisks indicate significant differences as determined by Dunnett’s test (*P < 0.05, **P < 0.01, and ***P < 0.001). Each replicate consists of five individual leaves. (**B**) Measurement of the stomatal aperture of Col-0–, *map4k1*-, *map4k2-1/2*–, and *MAP4K1-GFP*–overexpressing plants in the presence or absence of 5 μM ABA. The data were presented as box plots; box limits represent the first and third quartiles, with the medians marked as horizontal lines. Black dots indicate raw data points from the six individual leaves of each plant. The whiskers extend up to 1.5 times the interquartile range (IQR) from the first and third quartiles, with data points beyond this range displayed as outliers. Different letters indicate significant differences (Tukey’s test, *P* < 0.01). (**C**) Images show the representative stomata of Col-0–, *map4k1*-, *map4k2-1/2*–, and *MAP4K1-GFP*–overexpressing plants. The scale bar indicates 10 μm. (**D**) Water loss from detached leaves of Col-0, *map4k1map4k2-1*, and transgenic plants expressing FLAG-tagged MAP4K1 or its mutated forms (S479A, S479D, or K44N) in the *map4k1map4k2-1* background. Each replicate consists of five individual leaves. (**E**) Measurement of the stomatal aperture of Col-0, *map4k1map4k2-1*, and transgenic plants expressing FLAG-tagged MAP4K1 or its mutated forms in the presence or absence of 5 μM ABA. (**F**) Images show the representative stomata of Col-0, *map4k1map4k2-1*, and transgenic plants expressing FLAG-tagged MAP4K1 or its mutated forms. The scale bar indicates 10 μm.

To gain insight into the physiological roles of MAP4K1, we tested a transferred DNA (T-DNA) insertion mutant carrying a loss-of-function allele of *MAP4K1* (*map4k1; SALK_060372*) and transgenic plants overexpressing *MAP4K1-GFP* (*35Sp:MAP4K1-GFP*/Col-0) for ABA-related phenotypes (fig. S7, A to C). Water loss and stomatal aperture of these plants were measured to assess stomata function. The *map4k1* mutant exhibited slightly faster water loss and a larger stomatal aperture than Col-0 (Fig. 4, A to C), indicating that MAP4K1 is involved in the regulation of stomatal closure (Fig. 4, A and B). On the other hand, *35Sp:MAP4K1-GFP* exhibited the stomatal phenotype to levels similar to Col-0 (Fig. 4, A to C).

Next, double knockout mutants of MAP4K1 and MAP4K2, *map4k1map4k2-1* and *map4k1map4k2-2*, were generated to assess their functional redundancy (fig. S7, A and B). Compared to Col-0 or single knockout mutants, the rosette leaves of *map4k1map4k2-1* and *map4k1map4k2-2* double mutants showed a more pronounced shrinkage 6 hours after detachment (fig. S7D). Two hours after detachment, rosette leaves from *map4k1* had a 10% increase in water loss compared to Col-0, whereas *map4k1map4k2-1* and *map4k1map4k2-2* exhibited an ~20% increase in water loss (Fig. 4A). In support of this result, the aperture of stomata in *map4k1map4k2-1* and *map4k1map4k2-2* leaves was wider than that of stomata in Col-0 and *map4k1* leaves (Fig. 4, B and C). These results suggest that MAP4K1 and MAP4K2 function redundantly as positive regulators of ABA-induced stomatal closure.

On the other hand, the measurement of stomatal conductance indicated that ABA-induced stomatal closure in *map4k1map4k2-1* was not completely suppressed, unlike in *srk2e/ost1-3* (fig. S8, A and B). In addition, it showed that *map4k1map4k2-1* exhibited a response to CO_2_ similar to Col-0, in contrast to *srk2e/ost1-3* (fig. S8, C and D). These results suggest that MAP4K1/2 plays a partial role in ABA-induced stomatal closure but not in CO_2_ response.

In β-glucuronidase (GUS) reporter assays, *MAP4K1* and *MAP4K2* had similar tissue-specific expression patterns, including guard cells (fig. S9, A and B). Gene expression analysis revealed that *MAP4K1* and *MAP4K2* were expressed in both GCPs and MCPs (fig. S9C). In both MCPs and GCPs, the relative gene expression level of *MAP4K1* was much higher than that of *MAP4K2* (fig. S9C). *MAP4K1* was significantly induced by osmotic stress, while *MAP4K2* was not (fig. S9D). Similar results were observed in an ABA-deficient mutant *aao3*, suggesting that *MAP4K1/2* expression is not responsive to ABA (fig. S9D).

As described above, *MAP4K1* is expressed not only in guard cells but also in other plant tissues. Therefore, to clarify the guard cell–specific role of MAP4K1, we generated *map4k1map4k2-1* mutant lines complemented with *MAP4K1* driven by the *GC1 promoter*. As a result, *GC1p:MAP4K1* restored the ABA-induced stomatal closure of *map4k1map4k2-1* to the wild-type levels (fig. S10), suggesting that *MAP4K1* expression in guard cells plays a substantial role in ABA-induced stomatal closure.

### SRK2E/OST1–mediated phosphorylation of MAP4K1 at Ser^479^ is required for ABA-induced stomatal closure

To investigate the functional significance of phosphorylation of MAP4K1 at Ser^479^, we generated mutated forms of *MAP4K1* that code for MAP4K1 with Ala and Asp substitutions at Ser^479^. These Ser^479^→Ala (S479A) and S479D substitutions are expected to be nonphosphorylatable or phosphomimetic forms of MAP4K1, respectively. In addition, we generated a kinase-dead version of *MAP4K1* that codes for MAP4K1 with Asn substituted for Lys at position 44. The altered transgenes, *MAP4K1^S479A^*, *MAP4K1^S479D^*, and *MAP4K1^K44N^*, as well as *MAP4K1^WT^*, were stably expressed in *map4k1map4k2-1* plants (fig. S11).

As a result, the expression of *MAP4K1^WT^* or *MAP4K1^S479D^* suppressed the enhanced water loss of the phenotype of *map4k1map4k2-1* (Fig. 4D). In contrast, expression of *MAP4K1^S479A^* or *MAP4K1^K44N^* did not alter the enhanced water loss of *map4k1map4k2-1* (Fig. 4D). Consistent with these results, expression of *MAP4K1^WT^* or *MAP4K1^S479D^* rescued the ABA-induced stomatal closure of *map4k1map4k2-1*, but *MAP4K1^S479A^* and *MAP4K1^K44N^* did not (Fig. 4, E and F). These results suggest that the phosphorylation of MAP4K1 at Ser^479^ and MAP4K1 kinase activity play a substantial role in ABA-induced stomatal closure.

Furthermore, to explore the genetic interactions between SRK2E and MAP4K1/2, we generated *srk2emap4k1map4k2* triple mutants. These mutants exhibited water loss curves and stomatal apertures comparable to those of *srk2e/ost1-3* (fig. S12), suggesting that MAP4K1/2 operates as a component of the SRK2E/OST1–mediated pathway involved in ABA-induced stomatal closure.

### MAP4K1/2 is involved in ABA-induced [Ca^2+^]_cyt_ elevation

**Fig. 5.**
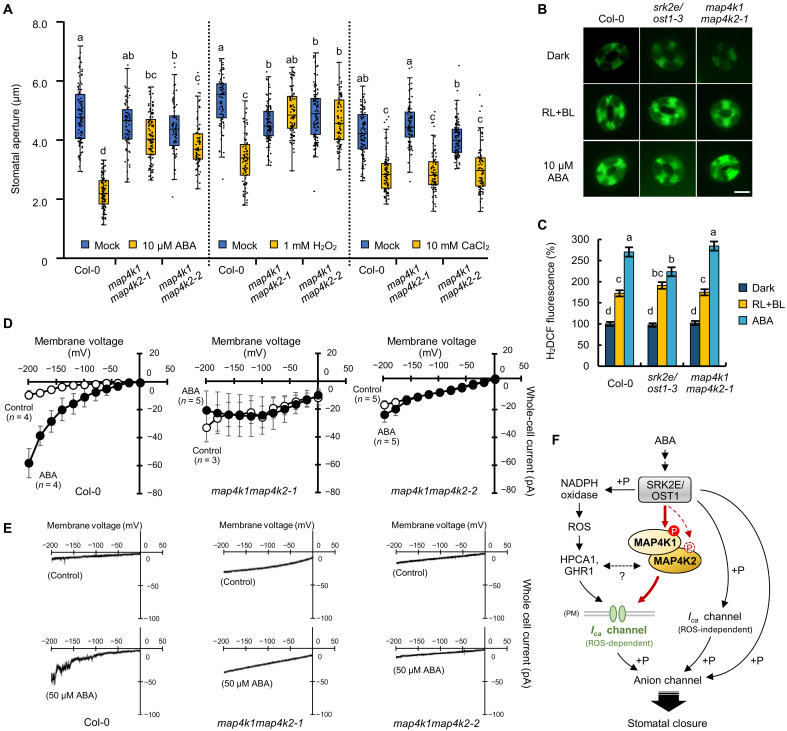
MAP4K1 and MAP4K2 are involved in the activation of plasma membrane Ca^2+^-permeable (*I*_Ca_) channels in ABA-induced stomatal closure. (**A**) Measurement of the stomatal aperture of Col-0, *map4k1map4k2-1*, and *map4k1map4k2-2*. Stomatal aperture was presented as box plots. Black dots indicate the raw data points from the six individual leaves of each plant. Different letters indicate significant differences (Tukey’s test, *P* < 0.01). (**B** and **C**) ROS accumulation in guard cells was indicated by the fluorescent dye H_2_DCFDA (B), and the relative ROS levels were quantified using ImageJ software (C). Epidermal strips were incubated in the dark or under RL and BL and then loaded with H_2_DCFDA. ABA (10 μM) was added, and reactions were carried out for another 30 min. The scale bar indicates 10 μm. Data represent the means ± SE (*n* = 75, pooled from triplicate experiments). Different letters indicate significant differences (Tukey’s test, *P* < 0.05). (**D**) Average current-voltage curves for ABA-activated *I*_Ca_ channels in GCPs isolated from Col-0, *map4k1map4k2-1*, and *map4k1map4k2-2*. The data represent the means ± SE. Open and closed circles indicate mock and 50 μM ABA treatment, respectively. ABA was added ~16 min after the start of whole-cell recording. Voltage was ramped from 0 to −198 mV at a ramp rate of 100 mV s^−1^. (**E**) Representative whole-cell Ca^2+^ current recordings from Col-0, *map4k1map4k2-1*, and *map4k1map4k2-2* GCPs. (**F**) Model of the SnRK2-MAP4K signaling module in the Ca^2+^-mediated pathway during ABA-induced stomatal closure. SRK2E/OST1 regulates both ROS-dependent and ROS-independent pathways to *I*_Ca_ channels and anion channels. ABA-activated SRK2E/OST1 directly phosphorylates Ser^479^ (Ser^488^) of MAP4K1 (MAP4K2) to regulate ABA-induced stomatal closure through activation of *I*_Ca_ channels.

SRK2E/OST1 regulates ABA-induced stomatal closure via ROS production, [Ca^2+^]_cyt_ elevation, and SLAC1 phosphorylation (*3*–*5*). To investigate whether MAP4K1/2 is required for these ABA-induced signaling events, we first applied H_2_O_2_ and Ca^2+^ to the epidermis of *Arabidopsis* leaves to mimic ROS production and [Ca^2+^]_cyt_ elevation, respectively. We then measured stomatal aperture in the treated leaves. Consistent with previous studies (*7*, *12*, *24*), ABA, H_2_O_2_, and Ca^2+^ induced stomatal closure in Col-0 leaves (Fig. 5A). In *map4k1map4k2*, stomatal closure in response to ABA and H_2_O_2_ was completely impaired but was normally induced by Ca^2+^ (Fig. 5A). This result suggested that MAP4K1/2 functions upstream of [Ca^2+^]_cyt_ elevation. ABA-responsive ROS production in guard cells was observed at a similar level in Col-0 and *map4k1map4k2-1* but reduced in *srk2e/ost1-3* (Fig. 5, B and C). The reduced response of *srk2e/ost1-3* was specific to ABA treatment, as similar levels of light-induced ROS production occurred in Col-0, *map4k1map4k2-1*, and *srk2e/ost1-3* (Fig. 5, B and C). These data suggest that MAP4K1/2 may not be involved in light- or ABA-responsive ROS production. Next, the relationship between MAP4K1/2 and SLAC1 was examined. Although MAP4K1/2 did not directly phosphorylate the N and C termini of SLAC1 in vitro (fig. S13A), phosphorylation of SLAC1 at Ser^86^, which is essential for SLAC1 activation (*25*, *26*), was significantly decreased in GCPs of *map4k1map4k2-1* (fig. S13, B and C). These results suggest that MAP4K1/2 affects SLAC1 phosphorylation in response to ABA but not through direct phosphorylation.

To measure ABA-dependent activation of plasma membrane Ca^2+^-permeable (*I*_Ca_) channels, whole-cell patch-clamp recording was performed using GCPs. In agreement with previous reports (*11*, *27*, *28*), externally applied ABA activated *I*_Ca_ channel currents in Col-0 GCPs (Fig. 5, D and E). However, ABA activation of *I*_Ca_ channels was not observed in *map4k1map4k2-1* or *map4k1map4k2-2* GCPs (Fig. 5, D and E). These results further support the involvement of MAP4K1/2 in ABA-responsive elevation of [Ca^2+^]_cyt_ in guard cells.

Our data support two possible models for how MAPK4K1/2 contribute to ABA-induced elevation of [Ca^2+^]_cyt_. First, MAP4K1/2 may function as an intermediary between SnRK2 and *I*_Ca_ channels. Alternatively, MAP4K1/2 may regulate SnRK2 activity, which in turn influences [Ca^2+^]_cyt_. To test the latter model, an in vitro phosphorylation assay was conducted using recombinant MAP4K1/2 and a kinase-dead form SRK2E^K50N^. In this assay, MAP4K1/2 did not phosphorylate SRK2E^K50N^ (fig. S14A). AtARK1/Raf4, an upstream protein kinase of SnRK2 (*29*–*31*), was used as a positive control. To examine the possibility that MAP4K1/2 indirectly affects SnRK2 activity, in-gel phosphorylation assays and 14-3-3 far-Western blot analyses using both seedlings and GCPs were performed. The levels of SnRK2 activity were similar between Col-0 and *map4k1map4k2-1* (fig. S14, B to E). Together, our results indicate that MAP4K1/2 functions downstream of SnRK2s and regulates [Ca^2+^]_cyt_ elevation via *I*_Ca_ channels.

## DISCUSSION

SRK2E/OST1, a member of subclass III SnRK2s, plays a critical role in ABA-induced stomatal closure, a process that is essential for plants to tolerate drought (*1*–*5*). To identify SnRK2 substrates, we did a phosphoproteomic and global proteomic analysis using *Arabidopsis* GCPs (Fig. 1A). The resulting dataset contains a total of 10,356 protein groups from Col-0, *srk2de*, and *abi1-1C* (table S1). This scale is comparable to a previous study on *Arabidopsis* guard cells (fig. S1A) (*22*). In addition, GO analysis confirmed that our data retained protein profiles that are known to occur in *Arabidopsis* guard cells (fig. S1D and table S4). Furthermore, guard cell–specific phosphoproteomics detected the ABA-dependent phosphorylation of several SnRK2 targets known to be involved in stomatal movement (Fig. 1D and table S3). These results suggest that our data are suitable for screening SnRK2 substrates in guard cells. In this regard, we successfully identified MAP4K1 as a substrate of SnRK2 through phosphoproteomics-based screening.

In general, to identify a protein (X) as a bona fide substrate of a protein kinase (Y), certain criteria should be met. For instance, the in vivo phosphorylation of X should be regulated under the presence of Y. In addition, Y can directly interact with and phosphorylate X in vivo or in vitro. In the case of MAP4K1, our phosphoproteomic analysis with *Arabidopsis* guard cells revealed that the ABA-dependent phosphorylation of MAP4K1 at Ser^479^ was abolished in *srk2de* and *abi1-1C* mutants (Fig. 2B). Subsequently, several lines of experiments showed that SRK2E/OST1 can phosphorylate Ser^479^ and directly interact with MAP4K1 in vitro or in vivo (Figs. 2, C to E, and 3, A to C). Thus, MAP4K1 meets the criteria to be considered a substrate phosphorylated by SnRK2. On the other hand, our phosphoproteomic analysis did not detect phosphopeptides derived from MAP4K2, and SnRK2 phosphorylated MAP4K2 only weakly in vitro (Fig. 2E and fig. S4C). Therefore, it remains unclear whether MAP4K2 is a substrate of SnRK2 (Fig. 5F).

Our genetic analyses revealed that MAP4K1 positively regulates ABA-induced stomatal closure in *Arabidopsis* (Fig. 4, A to C, and fig. S8) and that phosphorylation of Ser^479^ is required for MAP4K1 function (Fig. 4, D to F). Moreover, MAP4K1 genetically interacts with SRK2E (fig. S12). These findings suggest that a phosphosignaling pathway involving SnRK2 and MAP4K1 could be operational in response to ABA. In addition, MAP4K2 is the closest relative of MAP4K1 (fig. S2) and is functionally redundant with MAP4K1 in regulating ABA-induced stomatal closure (Fig. 4, A to C). SRK2E can directly interact with both MAP4K1 and MAP4K2 (Fig. 3, B and C), and MAP4K1 also interacts with MAP4K2 (Fig. 3, D and E). The shared interactions might be the cause of the redundancy between MAP4K1 and MAP4K2. Given that substitution of Ser^479^ did not affect MAP4K1 activity (fig. S15), additional phosphorylation events or other modifications beyond Ser^479^ may be required for MAP4K1 activation. At present, it is difficult to accurately describe the regulation of MAP4K1/2 kinase activity. Further studies will be required to elucidate the role of MAP4K1/2 phosphorylation in planta.

In *Arabidopsis*, there are 10 members of the MAP4K gene family (fig. S2), which are classified on the basis of their homology to yeast MAP4K/STE20, and some of them are involved in the response to environmental signals (*23*). For instance, SIK1 (SERIN/THREONINE KINASE 1)/MAP4K3 functions as a positive regulator in pattern-triggered immunity (*32*). MAP4K4/TARGET OF TEMPERATURE 3 (TOT3) physically interacts with MAP4K5/TOT3-INTERACTING PROTEIN 5 (TOI5) and MAP4K6/TOI4, and TOT3 functions redundantly with TOI5/TOI4 in hypocotyl/petiole elongation during thermomorphogenesis (*33*). Furthermore, BLUS1/MAP4K10 is directly phosphorylated by PHOT1/2 in a blue light–dependent manner, releasing BLUS1 from autoinhibition (*34*, *35*). Consistent with these reports, MAP4K1 and MAP4K2 also interact with each other and redundantly function during ABA-induced stomatal closure (Figs. 3, A, D, and E, and 4, A to C). Thus, our study supports the idea that the *Arabidopsis* MAP4K gene family including MAP4K1/2 may broadly function in plant environmental signaling.

Our data demonstrate that MAP4K1 and MAP4K2 are genetically required for ABA-induced increases in [Ca^2+^]_cyt_ and stomatal closure. The driving force behind stomatal closure is ion efflux, and one of the major regulatory pathways involves SnRK2 directly phosphorylating S-type anion channels including SLAC1 (*3*–*5*). Ca^2+^-dependent pathways are also involved in the regulation of SLAC1 (*28*). In this regard, ABA-induced ROS triggers an increase in [Ca^2+^]_cyt_ (*6*, *7*), which in turn activates CDPKs or CBLs-CIPKs to phosphorylate S-type anion channels (*27*, *36*). By directly measuring the activity of *I*_Ca_ channels, we found that MAP4K1/2 is required for ABA-induced increases in [Ca^2+^]_cyt_ (Fig. 5, D and E). The partial defect in ABA-induced stomatal closure observed in *map4k1map4k2* (Fig. 4) is consistent with the previous findings that ABA-induced [Ca^2+^]_cyt_ elevation is important for proper stomatal closure (*10*, *28*). ABA-responsive activation of *I*_Ca_ was largely impaired in *map4k1map4k2* (Fig. 5, D and E), whereas ABA-dependent ROS production occurred at normal levels in *map4k1map4k2* (Fig. 5, B and C). These data imply that MAP4K1/2 is not involved in ROS production but rather functions upstream of [Ca^2+^]_cyt_ elevation. Notably, the phosphorylation level of SLAC1 at Ser^86^ was significantly decreased in *map4k1map4k2-1* (fig. S13C). Recent studies have revealed that phosphorylation sites at the N terminus of SLAC1—specifically Ser^59^, Ser^86^, and Ser^120^—are important for its anion channel activity (*25*, *26*). Given that SnRK2 preferentially phosphorylates Ser^120^ of SLAC1 (*37*, *38*), MAP4K1/2 may regulate SLAC1 through a pathway distinct from the SnRK2-mediated direct phosphorylation of SLAC1 (Fig. 5F). Considering the partial effect of MAP4K1/2 on SLAC1 phosphorylation (fig. S13) and the observation that MAP4K1/2 is not involved in CO_2_ response (fig. S8), it can be inferred that the function of SnRK2-MAP4K is specific to the ROS-dependent Ca^2+^ pathway in ABA signaling. Together, our study proposes that the SnRK2-MAP4K signaling module regulates ABA-induced stomatal closure via a Ca^2+^-mediated pathway (Fig. 5F).

However, the mechanism(s) by which MAP4K1/2 regulates [Ca^2+^]_cyt_ and *I*_Ca_ is currently unknown. Recently, some (pseudo)protein kinases, such as GUARD CELL HYDROGEN PEROXIDE-RESISTANT1 (GHR1) and CARD1/HPCA1 (CANNOT RESPOND TO DMBQ1/H_2_O_2_-induced Ca^2+^ increases 1), have been proposed to be involved in the ROS-dependent regulation of [Ca^2+^]_cyt_ in guard cells (*39*–*42*). In addition, the ABA-activated *I*_Ca_ channels CNGC5/6/9/12 (CYCLIC NUCLEOTIDE–GATED CHANNEL 5/6/9/12) function in ROS-independent [Ca^2+^]_cyt_ elevation in guard cells, and Ser^27^ at the N terminus of CNGC6 is phosphorylated by SRK2E/OST1 (*43*, *44*). Therefore, it is likely that *I*_Ca_ channels in guard cells are regulated through both ROS-dependent and ROS-independent pathways (Fig. 5F). Notably, the *map4k1map4k2* phenotype in which stomatal closure was induced by exogenous Ca^2+^ but not by H_2_O_2_ (Fig. 5A) is more similar to *ghr1* and *hpca1* than to *cngc5/6/9/12* (*39*–*44*). Furthermore, the MAP4K1-GFP IP-MS analysis detected a few more peptides that belong to GHR1 or HPCA1 than the sample expressing GFP alone (table S5). In this regard, further studies will be required to clarify the relationship between MAP4K1/2 and the regulatory pathways of *I*_Ca_ channels.

Our results suggest that MAP4K1/2 likely functions not only in guard cells but also in other plant tissues. For example, *MAP4K1/2* is also expressed in leaves and roots (fig. S9, A and B), and *map4k1map4k2* seedlings showed ABA and osmotic stress insensitivity in cotyledon greening rate and stress-responsive gene expression (Fig. S16A,B), indicating that MAP4K1/2 is functional at this early stage of development. Furthermore, MAP4K1 Ser^479^ was phosphorylated in response to ABA and osmotic stress in *Arabidopsis* seedlings (fig. S16C). This result is consistent with previous reports from phosphoproteomics studies that investigated SnRK2 substrates in response to osmotic stress (*31*). Although MAP4K1 expressed in guard cells has been shown to play an important role in ABA-induced stomatal closure (fig. S10), it should also be considered that MAP4K1/2 in nonstomatal tissues may indirectly influence stomatal movement by altering cellular energy status, turgor pressure, etc. To date, reverse genetic analyses to identify the factors regulating stomatal movement have often focused on only genes highly expressed in guard cells. A gene like MAP4K1/2, which is widely expressed and functional in tissues other than guard cells, might have been overlooked by such a conventional approach.

In homologs of MAP4K1 in other plant species, Ser residues corresponding to Ser^479^ of MAP4K1 are well conserved among dicots, with the exception of legumes (fig. S17) (*45*)*.* This suggests that SnRK2-dependent phosphorylation of MAP4K1/2 may occur in a diversity of dicotyledonous crop plants. Further elucidation of MAP4K1/2 functions and signaling mechanisms will provide insights into ABA responses in plants and may provide avenues for future targeted engineering of drought tolerance or water use efficiency of crop species.

## MATERIALS AND METHODS

### Plant materials and growth conditions

*Arabidopsis thaliana* ecotype Columbia (Col-0) was used as wild-type plants. T-DNA insertion mutant lines, *map4k1* (SALK_060372), *map4k2-1* (SAIL_1255_G11), and *map4k2-2* (*SALK_115951*), were obtained from the Arabidopsis Biological Resource Center (ABRC). The double mutants *map4k1map4k2-1* and *map4k1map4k2-2* were generated by crossing *map4k1* and *map4k2*-*1* or *map4k2–2*. ABA response mutants, *srk2e/ost1-3, srk2de*, and *abi1-1C*, were obtained as previously described (*19*). Plant growth conditions were described in a previous study (*46*). To test responses to ABA or osmotic stress, seeds were sown on germination medium (GM) supplemented with or without indicated concentrations of ABA (Sigma-Aldrich) or mannitol (Wako), and cotyledon greening rates were recorded for 2 weeks after stratification according to a previous study (*46*).

### Isolation of *Arabidopsis* GCPs and ABA treatment

*Arabidopsis* GCPs were enzymatically isolated from rosette leaves of 4- to 5-week-old plants, following procedures outlined in previous studies (*17*, *18*). GCPs were adapted to the dark for 3 hours at 4°C, and then they were incubated in H^+^-pumping buffer [0.125 mM MES-NaOH (pH 6.0), 1 mM CaCl_2_, 0.4 M mannitol, and 10 mM KCl] under white light (90 μmol m^−2^ s^−1^) for 1 hour at 24°C. Samples were incubated with 10 μM ABA for the indicated periods (before adding ABA, it was described as “0 min”). The reaction was then stopped by adding trichloroacetic acid for far-Western blotting or by using liquid nitrogen for phosphoproteomic analysis, as described below.

### GST-14-3-3 far-Western blotting

Far-Western blotting analysis was performed using GST-tagged 14-3-3phi (GF14phi) protein as a probe according to a previous study with slight modifications (*20*, *21*). Briefly, precipitated GCP proteins were resuspended in sample loading buffer [62.5 mM tris-HCl (pH 6.8), 2% SDS, 5% sucrose, 2 ppm (parts per million) of bromophenol blue, and 100 mM dithiothreitol (DTT)] and separated on 10% SDS–polyacrylamide gel electrophoresis. After electrophoresis, the proteins were transferred to the polyvinylidene fluoride membrane (0.45 μm, Millipore) and then incubated with GST-14-3-3, which were detected using an anti-GST HRP conjugate antibody (RPN1236, Cytiva, MA).

### Vector constructions

The full-length cDNAs of *MAP4K1* and *MAP4K2* were cloned into the pENTR1A vector (Thermo Fisher Scientific) and confirmed by sequencing. Amino acid substitutions were carried out using site-directed mutagenesis as previously described (*47*). The cDNAs were then transferred into destination vectors, such as pGreen0029-GFP (*19*), R4pGWB 501 (*48*), pSITE-cEYFP-C1 (CD3-1649), or pSITE-cEYFP-N1 (CD3-1651), using Gateway LR Clonase II (Thermo Fisher Scientific). Upstream sequences (1300 or 1928 base pairs) of *MAP4K1* or *MAP4K2*, respectively, were amplified with specific primers with Gateway attB4/attB1r adaptor sequences and cloned into the pDONR P4-P1R vector (Thermo Fisher Scientific) using Gateway BP Clonase II (Thermo Fisher Scientific) to generate pDONR P4-P1R *MAP4K1p* and pDONR P4-P1R *MAP4K2p*. pDONR P4-P1R *MAP4K1p* or pDONR P4-P1R *MAP4K2p*, along with pENTR-GUS (Thermo Fisher Scientific), was combined with R4pGWB 501 (*48*) using Gateway LR Clonase II to generate R4pGWB 501 *MAP4K1p:GUS* and R4pGWB 501 *MAP4K2p:GUS*.

### Transgenic plants

The pGreen0029-GFP construct harboring *MAP4K1* was used to overexpress MAP4K1-GFP and prepared as described above. R4pGWB 501 constructs harboring *MAP4K1p:GUS*, *MAP4K2p:GUS*, and *MAP4K1p:MAP4K1^WT^/MAP4K1^S479A^/MAP4K1^S479D^/MAP4K1^K44N^-3xFLAG* were prepared as well. Each construct was transformed into Col-0 or *map4k1map4k2-1* with *Agrobacterium tumefaciens* strain GV3101 or GV3101 (with pSOUP). Transgenic plants were selected on GM containing Claforan (200 μg/ml) with kanamycin (50 μg/ml) or hygromycin (25 μg/ml). The expression of GFP-tagged or FLAG-tagged proteins was confirmed by immunoblotting using an anti-GFP polyclonal antibody (MBL, code no. 598) or anti-FLAG (DYKDDDDK) (Wako, 014-22383, lot SAR0168) as the primary antibody and using a horse anti-rabbit IgG antibody or horse anti-mouse IgG antibody (Vector Laboratories, CA) as the secondary antibody, respectively.

### Transient expression and subcellular localization analysis

Preparation of *Arabidopsis* MCPs and polyethylene glycol-calcium transfection were performed according to a previous study (*49*). For transient expression, pGreen0029-GFP constructs harboring *SRK2E/OST1* (*19*) or *MAP4K1* were prepared from *E. coli* (Takara, DH5α) using the QIAGEN Plasmid Midi Kit (QIAGEN) following the manufacturer’s protocol. For subcellular localization analysis, 10 μg of plasmid DNA was transfected into 5 × 10^4^ protoplasts, and GFP fluorescence was observed using a BX53 fluorescence microscope (Olympus).

### Phylogenetic analysis and prediction of kinase domains

*Arabidopsis* MAP4K1 orthologs were determined using BLASTP (Protein Basic Local Alignment Search Tool) search implemented in Phytozome version 13 (https://phytozome-next.jgi.doe.gov/) (*50*) against the specified organisms. Multiple alignments for the full-length protein sequence were generated by the MUSCLE algorithm (*51*) implemented in MEGA-X (*52*). The phylogenetic trees were built with the neighbor-joining method (*53*) on the basis of multiple alignments. All positions containing gaps were eliminated, and the phylogenies were evaluated using bootstrap with 1000 replicates (*54*). Prediction of kinase domains for each protein was performed using PROSITE (https://prosite.expasy.org/) (*55*).

### Histochemical GUS staining

*Arabidopsis* seedlings of *MAP4K1p:GUS* or *MAP4K2p:GUS* transgenic plants were pretreated with 90% cold acetone and then incubated in a GUS staining solution containing 1 mM 5-bromo-4-chloro-3-indolyl-β-d-glucuronic acid, 0.5 mM K_3_[Fe(CN)_6_], 0.5 mM K_4_[Fe(CN)_6_], 0.1% Triton X-100, and 50 mM sodium phosphate (pH 7.2) in the dark at 37°C overnight. The samples were washed and bleached with 70% ethanol. Images of the stained samples were captured using a microscope or an image scanner.

### Water loss analysis

One-week-old *Arabidopsis* plants were grown on GM agar plates under long-day conditions (16-hour light/8-hour dark) at 22°C and transferred to soil. The plants were then grown under the same conditions for an additional 3 weeks. The detached rosette leaves from ~4-week-old plants were placed on weighing dishes and left on the laboratory bench. Fresh weights were measured as previously described (*46*). Statistical analysis was conducted using Dunnett’s test with the R package.

### Measurement of stomatal aperture and stomatal conductance

Stomatal aperture in the abaxial epidermis was measured following a previous study with slight modifications (*56*). Briefly, epidermal strips were peeled from the rosette leaves of 4- to 5-week-old *Arabidopsis* seedlings grown on soil. The strips were then incubated in stomatal opening buffer [10 mM MES-KOH (pH 6.2), 5 mM KCl, and 0.1 mM CaCl_2_] under white light for 3 hours to fully expand the stomatal pore. Then, the strips were transferred to stomatal opening buffer containing the indicated concentrations of ABA for 2 hours. Stomatal apertures were photographed using a microscope and measured with ImageJ software. Statistical analysis was performed using Tukey’s post hoc test with the R package. Stomatal conductance was measured as previously described (*39*).

### RNA extraction and RT-qPCR analysis

Total RNA was extracted from 2-week-old seedlings, MCPs, or GCPs as described previously (*46*), and 500 ng of total RNA was used for reverse transcription using ReverTra Ace qPCR RT Master Mix with gDNA Remover (TOYOBO). RT-qPCR analysis was performed using LightCycler 480 SYBR Green I Master (Roche Life Science) with Light Cycler 96 (Roche Life Science). Each transcript was normalized by *GAPDH* and analyzed with three biological replicates. The gene-specific primers used for RT-qPCR are listed in table S6.

### Preparation of recombinant proteins

pMAL-c5X constructs harboring *SRK2E/OST1^K50N^* and *AtARK1/Raf4* cDNAs were prepared previously (*29*). *MAP4K1*, *MAP4K2*, and *SLAC1* cDNAs (N-terminal or C-terminal) were cloned in-frame into the pMAL-c5X vector (New England Biolabs) using the In-Fusion HD Cloning Kit (Takara Bio). Amino acid substitutions were introduced into pMAL-c5X MAP4K1 or MAP4K2 by site-directed mutagenesis to produce the kinase-dead form of *MAP4K1^K44N^* or *MAP4K2^K44N^* and the nonphosphorylatable form of *MAP4K1^K44N/S479A^* or *MAP4K2^K44N/S488A^*. The recombinant proteins were expressed and affinity purified from *E. coli* strain BL21 (DE3) using Amylose Resin (New England Biolabs), as previously described (*46*).

### In vitro phosphorylation assays

In vitro phosphorylation assay was performed as described previously (*46*). For LC-MS/MS analysis, proteins that underwent in vitro reactions were digested in 50 mM ammonium bicarbonate (NH_4_HCO_3_) with 0.2 μg of trypsin (Promega) at 37°C overnight. Tryptic digestion was stopped by adding trifluoroacetic acid to a final concentration of 1%. Peptides were desalted using an in-house C18 Stage-tip (*57*) made with a C18 Empore disk (CDS). Desalted peptides were dried using a centrifugal concentrator (CC-105, TOMY) and stored at −20°C until LC-MS/MS analysis.

### In-gel phosphorylation assays

In-gel phosphorylation assay was performed as previously described with slight modifications (*46*). Protein extraction buffer for 2-week-old *Arabidopsis* seedlings or isolated GCPs was modified as described below: 20 mM Hepes-KOH (pH 7.5), 0.5% Triton X-100, 100 mM NaCl, 5 mM MgCl_2_, 0.1 mM EDTA, 1 mM Na_3_VO_4_, 25 mM NaF, 50 mM β-glycerophosphate, 20% (v/v) glycerol, and 1% (v/v) protease inhibitor cocktail (Sigma-Aldrich).

### Sample preparation for IP-MS analysis

Crude was extracted from 2-week-old seedlings of *35Sp:GFP* and *35Sp:MAP4K1-GFP* transgenic plants using the protein extraction buffer as described in the above section. The mixture was centrifuged, and the supernatant was incubated with 20 μl of GFP selector for 1.5 hours at 4°C. After centrifugation, GFP selector beads were washed four times with the protein extraction buffer and once with 50 mM NH_4_HCO_3_. The reduction and alkylation mixture [10 mM tris(2-carboxyethyl)phosphine, 40 mM chloroacetamide, and 50 mM NH_4_HCO_3_] was added to the washed beads, followed by incubation at 24°C for 30 min in the dark. Then, 0.2 μg of trypsin (Promega) was added to the beads, followed by overnight incubation at 37°C. Tryptic digestion was stopped by adding trifluoroacetic acid to a final concentration of 1%. Peptides were desalted using an in-house C18 Stage-tip (*57*) made with a C18 Empore disk (CDS). Desalted peptides were dried using a centrifugal concentrator (CC-105, TOMY) and stored at −20°C until LC-MS/MS analysis.

### BiFC assay

BiFC assay was performed as previously described (*46*). Briefly, *A. tumefaciens* strain GV3101 (p19) expressing pSITE-nEYFP-N1:*SRK2E/OST1*, pSITE-cEYFP-N1:*MAP4K1/MAP4K2*, or pSITE-cEYFP-C1:*MAP4K1/MAP4K2* were mixed as indicated pairs and infiltrated into *N. benthamiana* leaves. After a few days, the complementary fluorescence of YFP (yellow fluorescent protein) was observed in epidermal cells using a BX53 fluorescence microscope (Olympus, Japan).

### In vitro pull-down assay

In vitro pull-down assay was performed as previously described (*47*). Briefly, *E. coli* cells expressing MBP, MBP-MAP4K1, MBP-MAP4K2, or GST-SRK2E/OST1 were homogenized with lysis buffer [50 mM tris-HCl (pH 7.4), 150 mM NaCl, 1% NP-40, and 0.25% sodium deoxycholate (SDC)]. Lysates containing GST-SRK2E/OST1 or MBP-tagged proteins were mixed, and a portion of the mixture was set aside as input fraction. Amylose resin beads (New England Biolabs, MA) were added to the mixture and incubated for 3 hours at 4°C. The beads were washed four times with lysis buffer. Proteins were then eluted from the beads using lysis buffer containing 10 mM maltose. Input and eluted fractions were detected by Western blotting.

### Sample preparation for phosphoproteomic/proteomic analysis

Protein extraction and digestion were performed according to a previous study with some modifications (*58*). Briefly, total protein lysates were extracted from isolated GCPs in protein extraction buffer [100 mM tris-HCl (pH 9.0) and 6 M guanidine hydrochloride], followed by heating of the lysates for 5 min at 95°C. The lysate samples were placed on ice, and the samples were sonicated using a microultrasonic homogenizer (Q125, QSONICA, US) and centrifuged at 17,400*g* for 20 min at 4°C. The supernatant was then precipitated by the methanol-chloroform method, and the protein pellets were resuspended in a digestion buffer [100 mM tris-HCl (pH 9.0), 12 mM sodium lauryl sulfate (SLS), and 12 mM SDC]. Two hundred micrograms of protein per sample was incubated in a reduction and alkylation mixture [10 mM tris(2-carboxyethyl)phosphine, 40 mM chloroacetamide, and 50 mM ammonium bicarbonate] for 30 min at 24°C in the dark. After a fivefold dilution with 50 mM ammonium bicarbonate, proteins were digested overnight at 37°C with 2 μg of trypsin (Promega). Phase transfer surfactants (such as SLS and SDC) were removed according to a previous study (*59*). After removal, 10% of the total volume of peptide samples was set aside for global proteomic analysis, and phosphopeptides were enriched using hydroxy acid–modified metal oxide chromatography, as described in previous studies (*60*, *61*). Digested peptides or enriched phosphopeptides were desalted using an in-house Stage-tip made with SDB Empore disks (CDS). After desalting, the phosphopeptides were dried and stored at −20°C until LC-MS/MS analysis.

### LC-MS/MS analysis and raw data processing

Prepared peptide samples were analyzed using a nano-LC system, Easy-nLC 1200 (Thermo Fisher Scientific), connected in line to a quadrupole-Orbitrap mass spectrometer, Orbitrap Exploris 480 (Thermo Fisher Scientific), equipped with an aerodynamic high-field asymmetric waveform ion mobility spectrometry (FAIMS) device, FAIMS Pro (Thermo Fisher Scientific). Dried peptides were resuspended in a solution of 2% (v/v) acetonitrile with 0.1% (v/v) formic acid (FA) and then loaded directly into a C18 nano-HPLC capillary column (NTCC-360/75-3, 75-μm inside diameter by 15-cm length, Nikkyo Technos). Peptides were eluted at 300 nl/min at 60°C with a nonlinear gradient program for 140 min for phosphoproteomic analysis. IP-MS and in vitro kinase samples were eluted for 105 min. The mobile phase buffer consisted of 0.1% FA (buffer A) with an elution buffer of 0.1% FA in 80% acetonitrile (buffer B). The 140-min program was executed under the following conditions: 0 to 5 min, 6% B held; 5 to 79 min, 6 to 23% B; 79 to 107 min, 23 to 35% B; 107 to 125 min, 35 to 50% B; 125 to 130 min, 50 to 90% B; 130 to 140 min, 90% B held. The 105-min program was executed as follows: 0 to 54 min, 6 to 23% B; 54 to 75 min, 23 to 35% B; 75 to 90 min, 35 to 50% B; 90 to 95 min, 50 to 65% B; 95 to 105 min, 65% B held. Eluted peptides were ionized at a source voltage of 2.2 kV and detected using data-dependent acquisition in positive ion mode. MS1 spectra were collected in the range of 375 to 1500 *m*/*z* (mass/charge ratio). For phosphoproteomic analysis, the resolving power was set to 60,000. For IP-MS and in vitro kinase assay, the resolving power was set to 120,000. For all analyses, MS2 spectra were collected in the range above 120 *m*/*z* at a resolving power of 30,000. In the FAIMS compensation voltage (CV), two conditions were used (−40/−60 CV or −50/−70 CV), and the resolution was set to “Standard” (inner temperature of 100°C/outer temperature of 100°C) under both conditions.

For phosphoproteomic or proteomic analysis and in vitro kinase assay, peptide/protein identification and MS1-based label-free quantification (LFQ) were performed using Proteome Discoverer 2.5 (PD2.5) (Thermo Fisher Scientific). For IP-MS analysis, peptide/protein identification was carried out using PD3.1 (Thermo Fisher Scientific). MS/MS spectra were searched using SEQUEST HT (PD2.5) or CHIMERYS (PD3.1) algorithms against the *Arabidopsis* protein database (Araport11_genes.201606.pep.fasta). In both of search engines, the following search parameters were used as follows: [Digestion Enzyme; trypsin], [Maximum Missed Cleaves; 2], [Peptide Length; 6-144], [Precursor Mass Tolerance; 10 ppm], [Fragment Mass Tolerance; 0.02 Da], [Static Modification; cysteine (C) carbamidomethylation], [Variable Modification; methionine (M) oxidation/N-terminal acetylation/serin (S) threonine (T) tyrosine (Y) phosphorylation], [Maximum Variable Modifications; 3]. Peptide validation was performed using the Percolator algorithm, and only high-confidence peptides [false discovery rate (FDR) < 1%] were used for protein inference and MS1-based LFQ. For phosphoproteomic analysis, the site localization probability of phosphorylated amino acid was calculated using the IMP-ptmRS node implemented in Proteome Discoverer software.

To calculate the abundance fold change for different biological conditions, the phosphopeptides with more than or equal to three quantitative values in each sample group were selected (the number of 14,645 peptide groups in table S3). The abundances were normalized by Levenberg-Marquardt optimization (*62*), and missing values were replaced by the minimum value. The source code was described in a previous study (*46*).

### Analysis of proteomic data

Unless otherwise noted, basic calculations related to proteomic data were performed in the Excel program. For visualization of phosphopeptide intensities as a heatmap, mean values for each peptide were scaled to 100 (table S7). GO enrichment analysis was performed as follows: The protein IDs in each Excel sheet (nos. 1, 4, and 7 in table S3) were uploaded to PANTHR (www.pantherdb.org/), and the background database was set as *Arabidopsis* all-gene IDs. The GO terms shown in the figures were selected as GO terms with less than 1% FDR.

### Measurement of ABA-induced ROS production in guard cells

The measurement of ROS levels in guard cells was performed according to a previous study (*56*). Briefly, dark-adapted epidermal strips were incubated in stomatal opening buffer [5 mM MES-bistrispropane (pH 6.5), 50 mM KCl, and 0.1 mM CaCl_2_] for 1.5 hours in the dark or in red light (RL) (50 μmol m^−2^ s^−1^) and blue light (BL) (10 μmol m^−2^ s^−1^) at 24°C and then treated with 50 μM 2′,7′-dichlorodihydrofluorescein diacetate (H_2_DCFDA) for 30 min. After washing the epidermis to remove excess dye, ABA-induced ROS production was measured by adding 10 μM ABA under the RL and BL, and the epidermis was incubated for another 30 min. 2,7-Dichlorofluorescein diacetate fluorescence was observed using fluorescence microscopy (Eclipse TS100; Nikon), and the fluorescence intensity was measured using ImageJ software.

### Whole-cell patch-clamp measurement of *Arabidopsis* GCPs

*I*_Ca_ channel currents in *Arabidopsis* GCPs were recorded according to previous studies (*27*, *63*). Whole-cell currents were recorded using a MultiClamp 700B patch-clamp amplifier (Molecular Devices, San Jose, CA), and the data were analyzed with pCLAMP 10.3 software (Molecular Devices). The pipette solution contained 10 mM BaCl_2_, 0.1 mM DTT, 5 mM NADPH, 4 mM EGTA, and 10 mM Hepes-tris (pH 7.1). The bath solution contained 100 mM BaCl_2_, 0.1 mM DTT, and 10 mM MES-tris (pH 5.6). The osmolarity of the solutions was adjusted with d-sorbitol to 500 mmol kg^−1^ (pipette) and 485 mmol kg^−1^ (bath).

### Accession numbers

Sequence data for the genes described here are available at TAIR (www.arabidopsis.org/) under the following accession numbers: MAP4K1/AtMAP4Kα1 (AT1G53165), MAP4K2/AtMAP4Kα2 (AT3G15220), MAP4K3/SIK1 (AT1G69220), MAP4K4/TOT3 (AT5G14720), MAP4K5/TOI5 (AT4G24100), MAP4K6/TOI4 (AT4G10730), MAP4K7/TOI3 (AT1G70430), MAP4K8/TOI1 (AT1G79640), MAP4K9/TOI2 (AT1G23700), MAP4K10/BLUS1 (AT4G14480), and SLAC1 (AT1G12480).
