# Supplementary material for: MAP4K1 and MAP4K2 regulate ABA-induced and Ca2+-mediated stomatal closure in Arabidopsis
Source: Sci Adv. 2025 Dec 19;11(51):eadt4916. doi: 10.1126/sciadv.adt4916 (PMC12716388; doi:10.1126/sciadv.adt4916)
Supplement: Supplementary file 1 — Figs. S1 to S17 Legends for tables S1 to S7 [file sciadv.adt4916_sm.pdf]

Science Advances
AAAS

Supplementary Materials for

MAP4K1 and MAP4K2 regulate ABA-induced and Ca^{2+}-mediated stomatal closure in *Arabidopsis*

Kota Yamashita *et al.*

Corresponding author: Taishi Umezawa, taishi@cc.tuat.ac.jp

Sci. Adv. **11**, eadt4916 (2025)
DOI: 10.1126/sciadv.adt4916

The PDF file includes:

Figs. S1 to S17
Legends for tables S1 to S7

Other Supplementary Material for this manuscript includes the following:

Tables S1 to S7

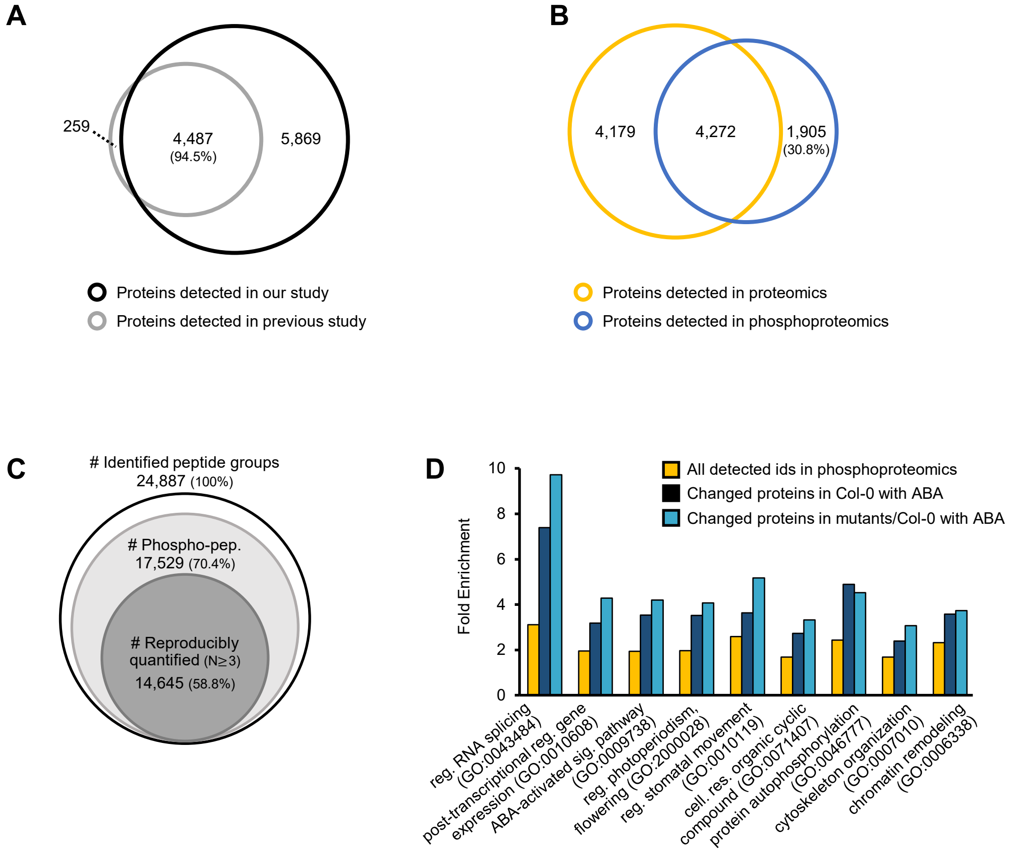

Figure S1. An overview of phosphoproteomic analysis for Arabidopsis guard cell protoplasts. **A**, An overlap between the proteins detected in our study and a previous study (22). **B**, An overlap between the proteins detected in proteomic and phosphoproteomic analysis in this study. **C**, The percentage of detected and quantified phosphopeptides. The phosphopeptides with ≥ three quantitative values in each sample group were "Reproducibly quantified". **D**, Gene ontology (GO) analysis of proteins with significant changes in ABA or in mutants. GO terms were evaluated by PANTHR program and with less than 1% FDR were employed.

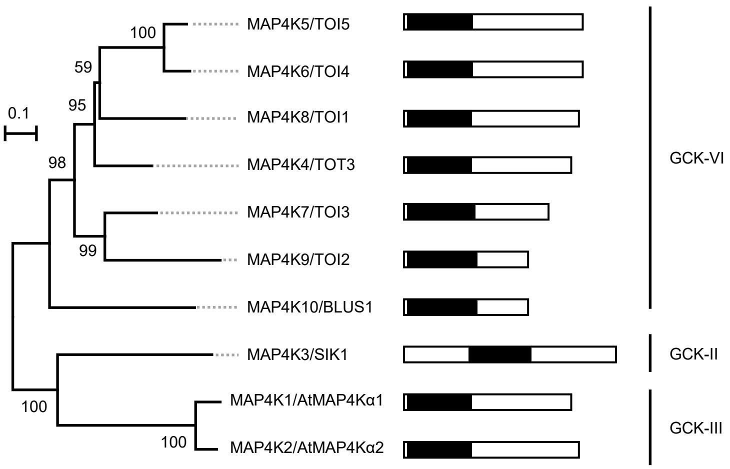

Figure S2. A phylogenetic tree of the MAP4K family in Arabidopsis. A schematic of the protein sequences is shown in right, including the position of kinase domain (black). Arabidopsis MAP4Ks are classified to germinal center kinase (GCK)-type II, III or VI based on mammalian proteins.

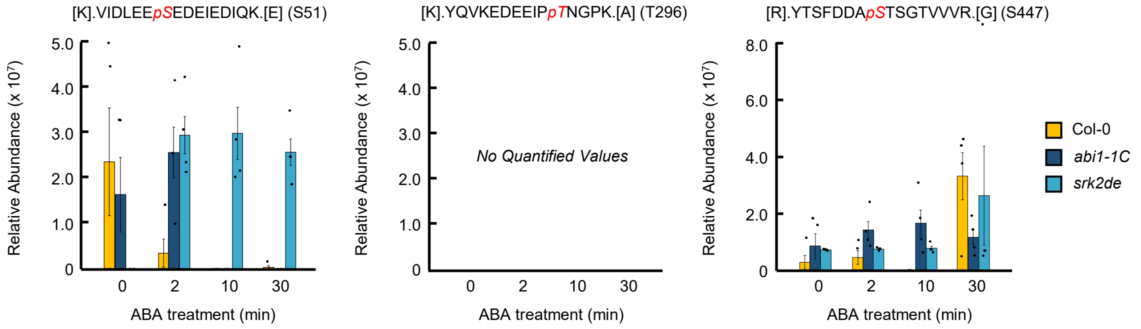

Figure S3. Relative abundance of MAP4K1 peptides containing phosphorylated Ser-51, Thr-296, or Ser-447. Data represent means ± SE (n = 4, biologically independent replicate).

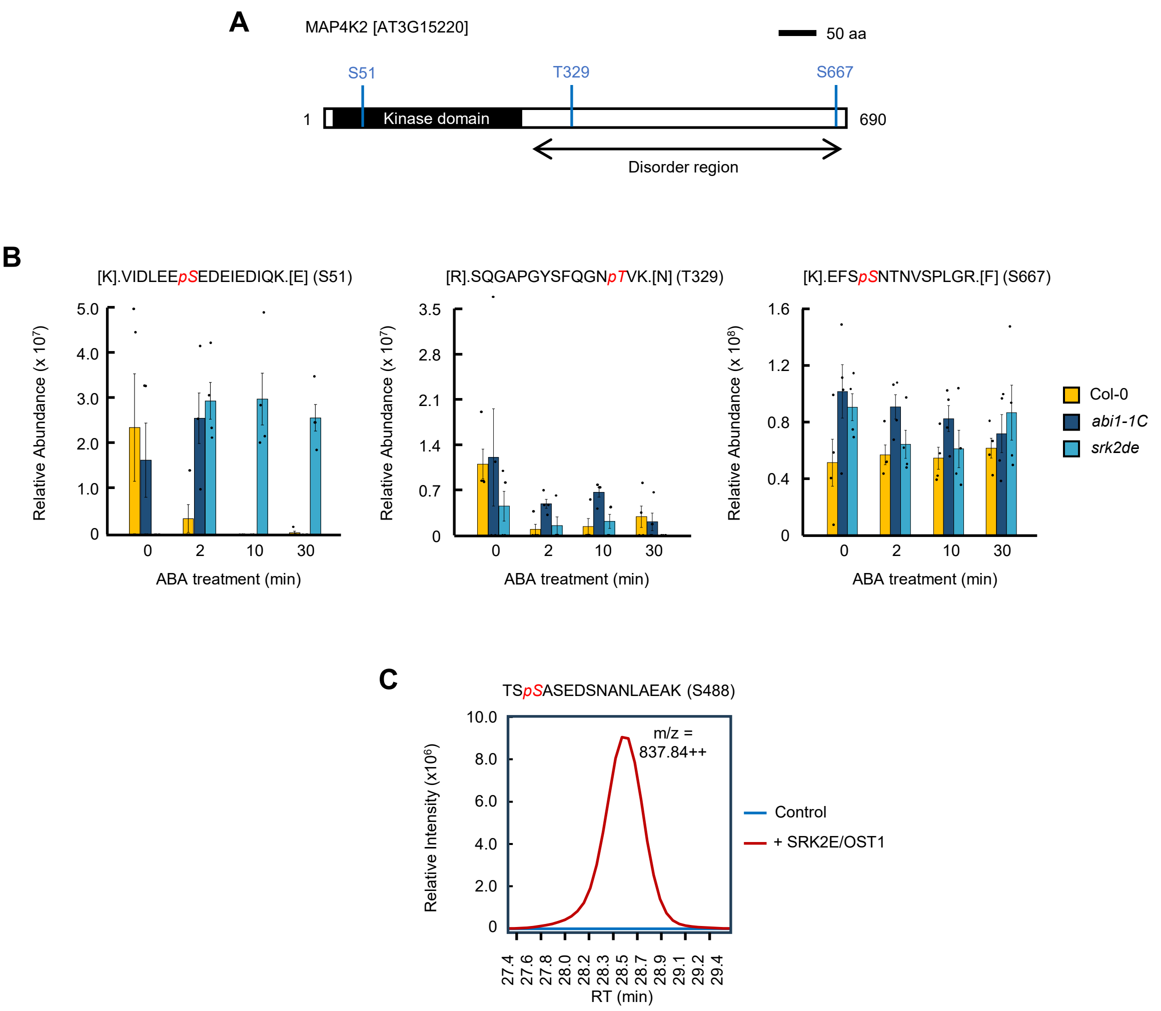

Figure S4. Phosphorylation sites of MAP4K2 detected in this study.

A, Domain structure of MAP4K2. Phosphorylation sites identified in this study were shown in blue. **B**, Relative abundance of MAP4K2 peptides containing phosphorylated Ser-51, Thr-329, or Ser-667. Data represent means ± SE (n = 4, biologically independent replicate). **C**, SRK2E/OST1 phosphorylates MAP4K2 at Ser-488 *in vitro*. LC-MS/MS analysis detected a phosphopeptide containing Ser-488 following an *in vitro* phosphorylation assay using SRK2E and MAP4K2.

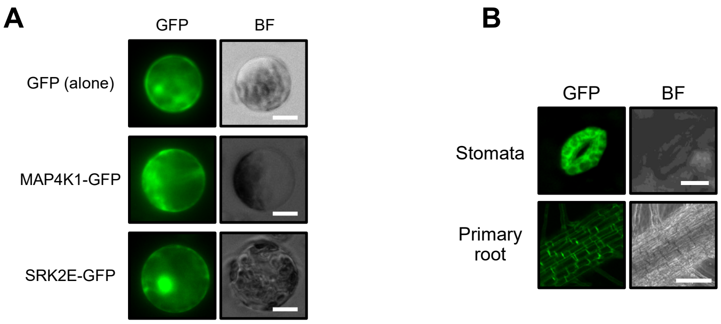

Figure S5. Subcellular localization of MAP4K1-GFP.
A, GFP, MAP4K1-GFP and SRK2E/OST1-GFP were transiently expressed in Arabidopsis mesophyll cell protoplasts. Scale bars indicate 25 µm. **B**, GFP fluorescence in *35Sp:MAP4K1-GFP* transgenic plants. Scale bars indicate 15 µm or 100 µm for the images of stomata or primary root, respectively.

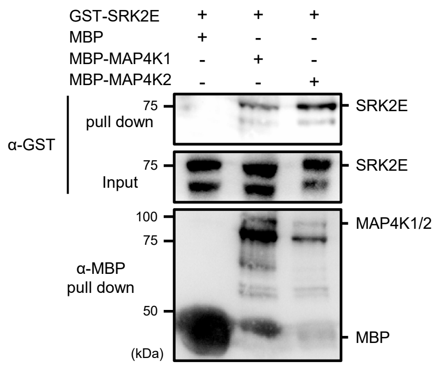

Figure S6. *in vitro* pull-down assay for MBP-MAP4K1/2 and GST-SRK2E. GST tagged SRK2E/OST1 and MBP tagged MAP4K1/2 were detected by immunoblotting using an anti-GST antibody and an anti-MBP antibody, respectively.

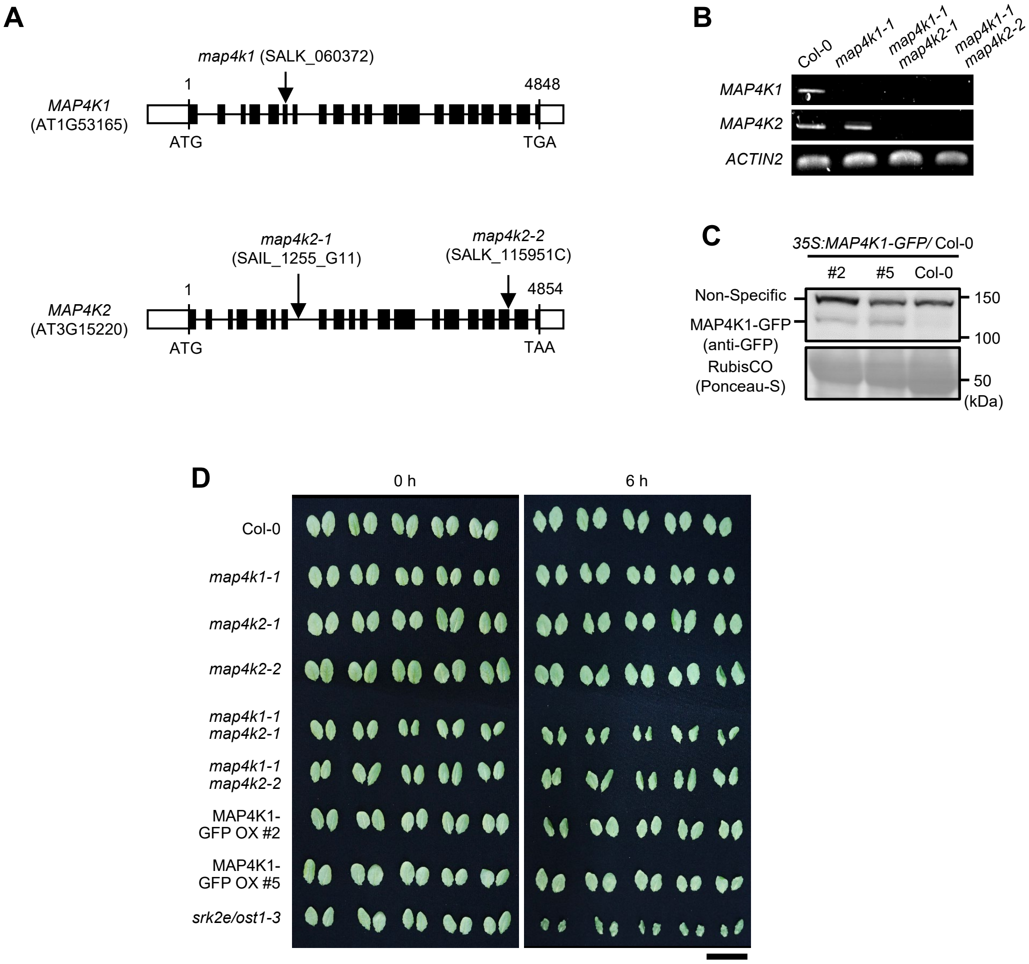

Figure S7. *MAP4K1/2* knockout mutants and *MAP4K1* overexpressing plants.
A, T-DNA insertions in *map4k1*, *map4k2-1* and *map4k2-2*. Solid boxes and lines indicate exon and intron, respectively. **B**, RT-PCR analysis of Col-0, *map4k1*, *map4k2-1* and *map4k2-2*. ACTIN2 was used as a positive control. **C**, Western blot analysis of *35Sp:MAP4K1-GFP* plants. **D**, A leaf wilting test was conducted using the following genotypes: Col-0, *map4k1*, *map4k2-1*, *map4k2-2*, *map4k1map4k2-1*, *map4k1map4k2-2*, *MAP4K1-GFP OX* #2/#5, and *srk2e/ost1-3*. Photos were taken at 0 h or 6 h after detached. The scale bar indicates 3 cm.

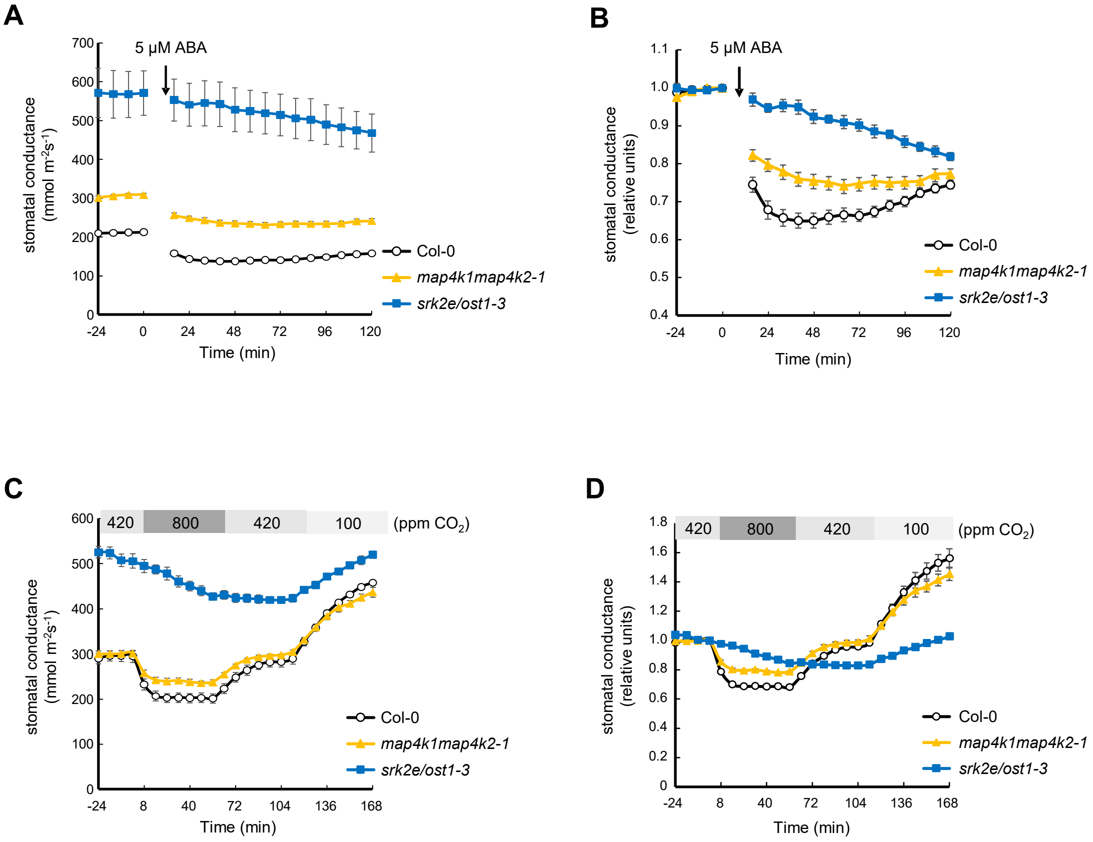

Figure S8. Stomatal conductance in response to ABA and CO_2.
A, Stomatal conductance in response to ABA was measured for Col-0, *map4k1map4k2-1* and *srk2e/ost1-3*. The arrow indicates the time point of ABA treatment. B, Stomatal conductance in response to ABA, expressed in relative units. **C**, Stomatal conductance in response to CO_2 was measured for Col-0, *map4k1map4k2-1* and *srk2e/ost1-3*. The CO_2 concentration was controlled as indicated. **D.** Stomatal conductance in response to CO_2, expressed in relative units.

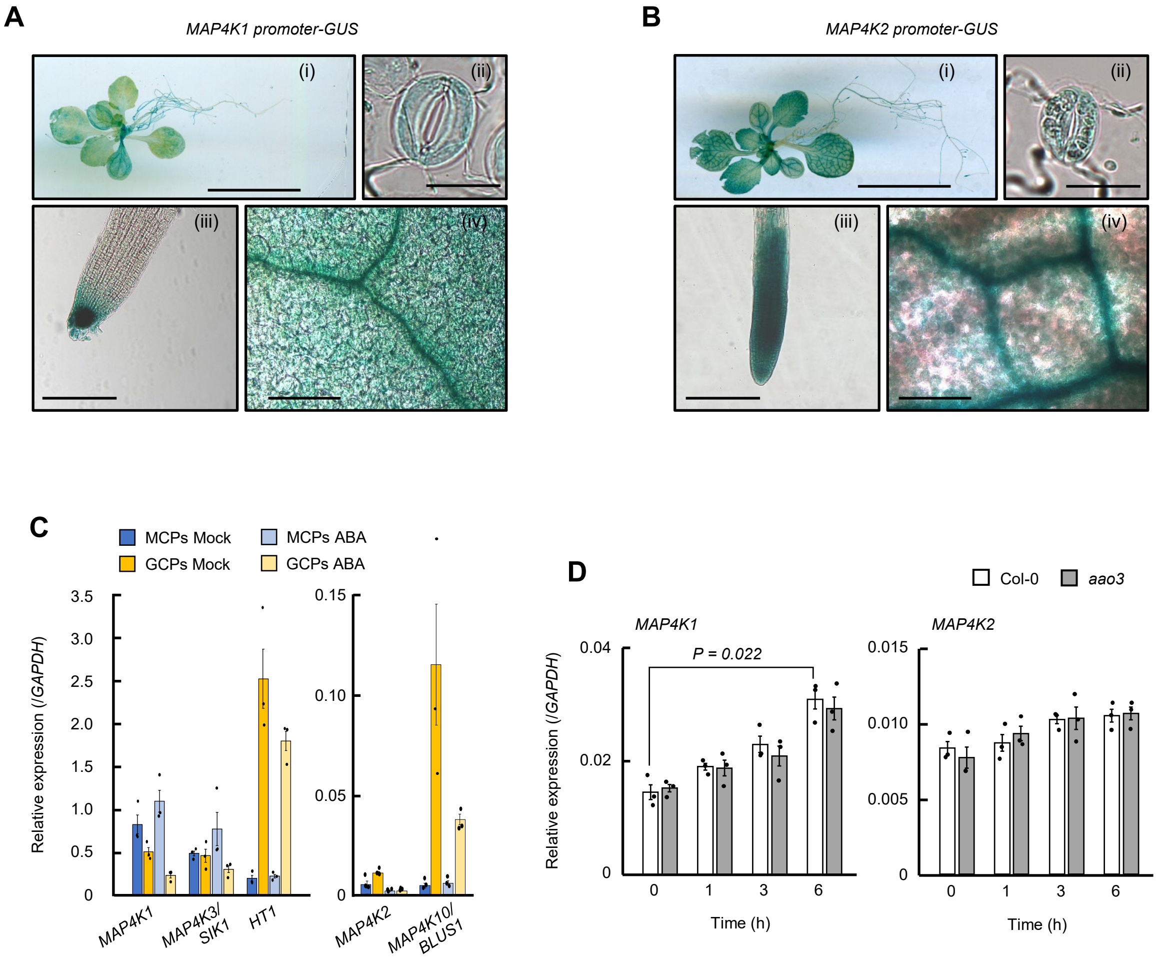

Figure S9. Expression patterns of MAP4K genes.

A and B, A histochemical GUS staining of *MAP4K1 promoter-GUS* (**A**) and *MAP4K2 promoter-GUS* plants (**B**). Each panel displays whole plants (i), stomata (ii), root tips (iii), and leaf veins (iv), respectively. Each scale bar indicates 1 cm (i), 10 µm (ii), 40 µm (iii) and 100 µm (iv) , respectively. **C**, A quantitative RT-PCR analysis for *MAP4K1*, *MAP4K2*, *MAP4K3/SIK1* and *MAP4K10/BLUS1*. Mesophyll cell protoplasts (MCP) and guard cell protoplasts (GCP) were prepared from Col-0 plants treated with/without ABA for 30 min. **D**, A quantitative RT-PCR analysis for *MAP4K1* and *MAP4K2* in Col-0 and *aao3* treated with 400 mM sorbitol for indicated time periods. Bars indicate means ± SE (n = 3), and significance was determined using two-tailed Student's t-test adjusted with Bonferroni.

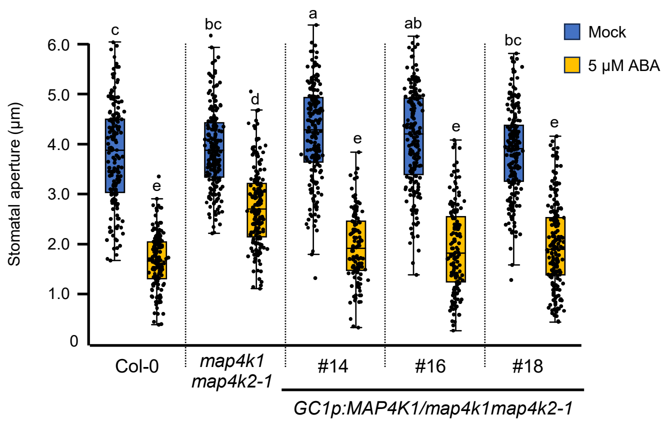

Figure S10. Guard cell-specific expression of MAP4K1 can restore *map4k1map4k2* stomatal phenotype. Measurement of the stomatal aperture of Col-0, m*ap4k1map4k2-1* and *GC1p:MAP4K1* in the presence or absence of 5 µM ABA. The data was presented as box plots; box limits represent the first and third quartiles, with the medians marked as horizontal lines. Black dots indicate raw data points from the six individual leaves of each plant. The whiskers extend up to 1.5 times the interquartile range (IQR) from the first and third quartiles, with data points beyond this range displayed as outliers. Different letters indicate significant differences (Tukey's test, $P < 0.01$).

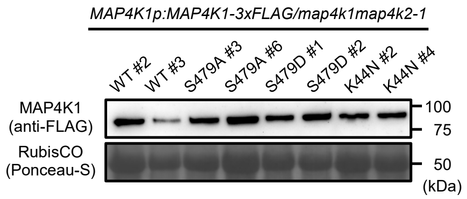

\

Figure S11. Western blotting showing the expression of MAP4K1 WT/S479A/S479D/K44N in *map4k1map4k2-1*. Crude extracted from 2-weeks old transgenic plants of *MAP4K1p:MAP4K1* $^{WT/S479A/S479D/K44N}$ *-3xFLAG* were subjected to western blotting analysis.

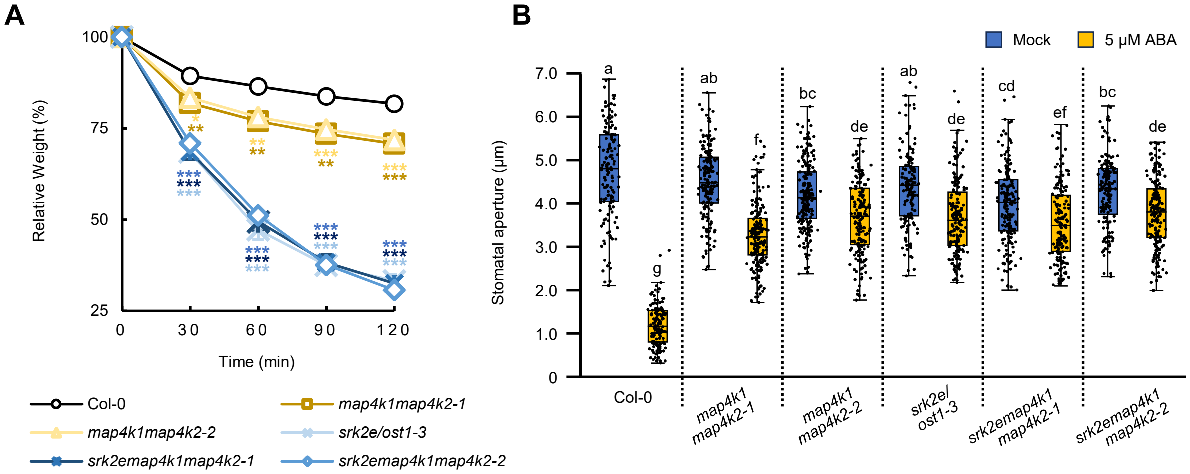

Figure S12. Genetic analysis of stomatal phenotype in *srk2emap4k1map4k2* triple knockout mutants.

A, Water loss from detached leaves of Col-0 as wild-type, m*ap4k1map4k2-1*, m*ap4k1map4k2-2*, *srk2e/ost1-3*, *srk2emap4k1map4k2-1* and *srk2emap4k1map4k2-2*. Data are means ± SE (n = 6), and asterisks indicate significant differences as determined by Dunnett's test (**P* < 0.05, ***P* < 0.01, ****P* < 0.001). Each replicate consists of five individual leaves. **B,** Measurement of the stomatal aperture of Col-0, m*ap4k1map4k2-1*, m*ap4k1map4k2-2*, *srk2e*, *srk2emap4k1map4k2-1* and *srk2emap4k1map4k2-2* in the presence or absence of 5 μM ABA. The data was presented as box plots; box limits represent the first and third quartiles, with the medians marked as horizontal lines. Black dots indicate raw data points from the six individual leaves of each plant. The whiskers extend up to 1.5 times the interquartile range (IQR) from the first and third quartiles, with data points beyond this range displayed as outliers. Different letters indicate significant differences (Tukey's test, P < 0.01).

\

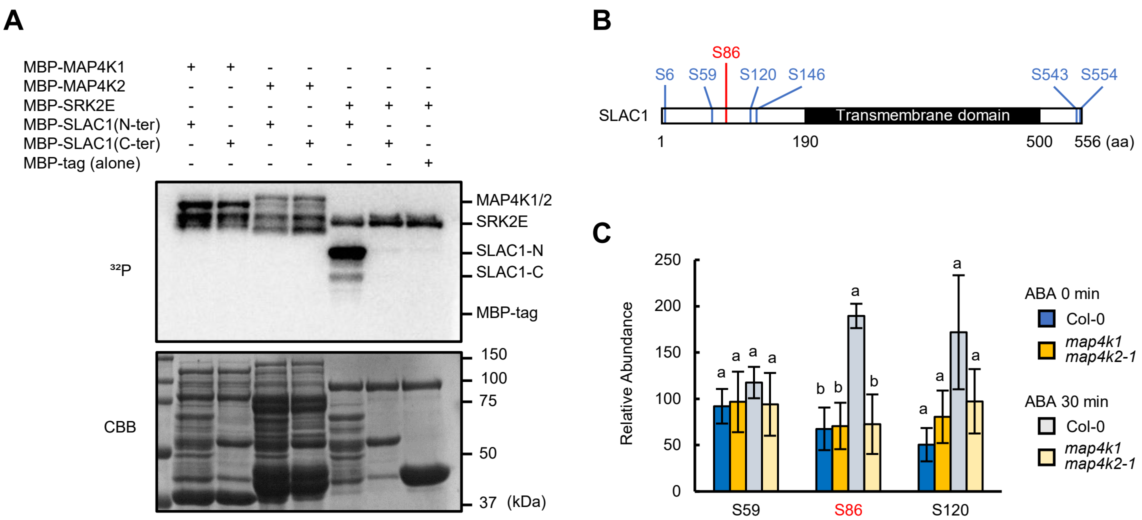

Figure S13. MAP4K1/2 indirectly influences the phosphorylation of SLAC1.
A, An *in vitro* phosphorylation assay was conducted using MBP-MAP4K1, -MAP4K2, -SRK2E/OST1. MBP-SLAC1(N-ter), -SLAC1(C-ter) and MBP were used as substrates. Phosphorylation levels were detected through autoradiography (^{32}P). **B**, Domain structure of SLAC1 with numbers indicating the phosphorylation sites detected in this study. **C**, The phosphorylation levels of Ser-59, Ser-86 and Ser-120 of SLAC1. Quantitative data for each phosphopeptide were obtained from LC-MS/MS analysis for Col-0 and *map4k1map4k2-1* GCPs treated with/without ABA for 30 min. Data represent means ± SE (n = 4, biologically independent replicate). Different letters indicate significant differences (Tukey's test, $P < 0.05$).

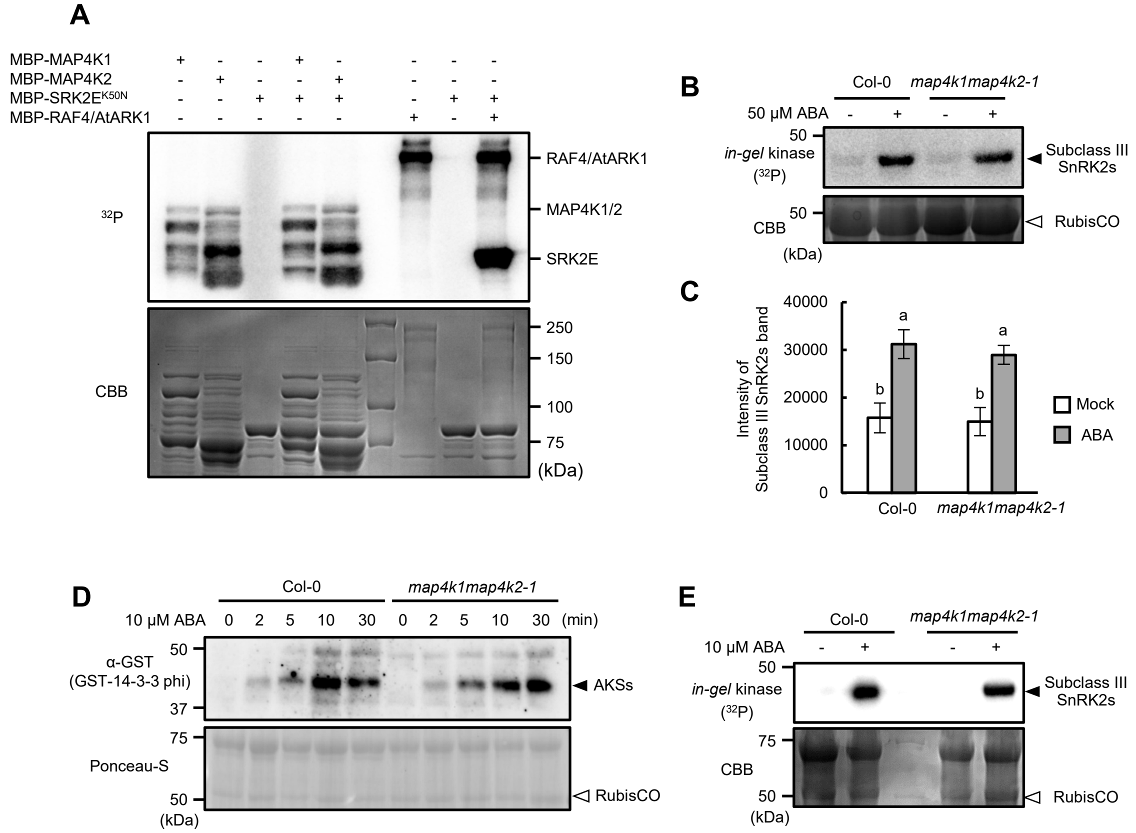

Figure S14. MAP4K1/2 does not influence ABA-dependent activation of SnRK2s.
A, An *in vitro* phosphorylation assay was conducted using MBP-MAP4K1, -MAP4K2, and -RAF4/AtARK1, with MBP-SRK2E^{K50N} serving as the substrate. Phosphorylation levels were detected through autoradiography (^{32}P). Coomassie Brilliant Blue (CBB)-staining shows protein loading in each lane. **B and C**, SnRK2 activity in Col-0 and *map4k1map4k2-1* plants treated with/without ABA. An in-gel phosphorylation assay was performed using crude extracts from Col-0 and *map4k1map4k2-1* seedlings. The black arrow indicates SnRK2 activity detected by autoradiography (**B**), and band intensity was measured using the ImageJ program (**C**). Data are presented as means ± SE (n = 4) and different letters in indicate significant differences (Tukey's test, $P < 0.05$). The open arrow indicates RubisCO in a CBB-stained gel. **D**, Far-western blotting analysis of 14-3-3 protein using crude extracts from Col-0 and *map4k1map4k2-1* GCPs treated with/without 10 µM ABA for indicated time periods. GST-tagged 14-3-3 (GF14 phi) protein was used as a probe. Black arrows and open arrows indicate the positions of ABA-responsive Kinase Substrates (AKSs) and RubisCO, respectively. This experiment was repeated two times biologically independent with similar results. **E**, An in-gel phosphorylation assay was conducted using crude extracts from Col-0 and *map4k1map4k2-1* GCPs treated with 10 µM ABA for 10 min. Black and open arrows indicate SnRK2 activity and RubisCO, respectively. This experiment was repeated two times biologically independent with similar results.

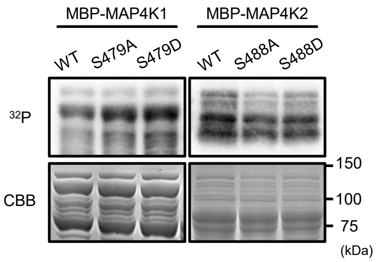

Figure S15. Effects of amino acid substitutions at Ser479/488 on the autophosphorylation of MAP4K1/2.

MBP-MAP4K1$^{WT/S479A/S479D}$ and MBP-MAP4K2$^{WT/S488A/S488D}$ were subjected to an *in vitro* phosphorylation assay in the presence of [γ-^{32}P]ATP. Autophosphorylation levels were detected through autoradiography (^{32}P). CBB staining showed protein loading in each lane.

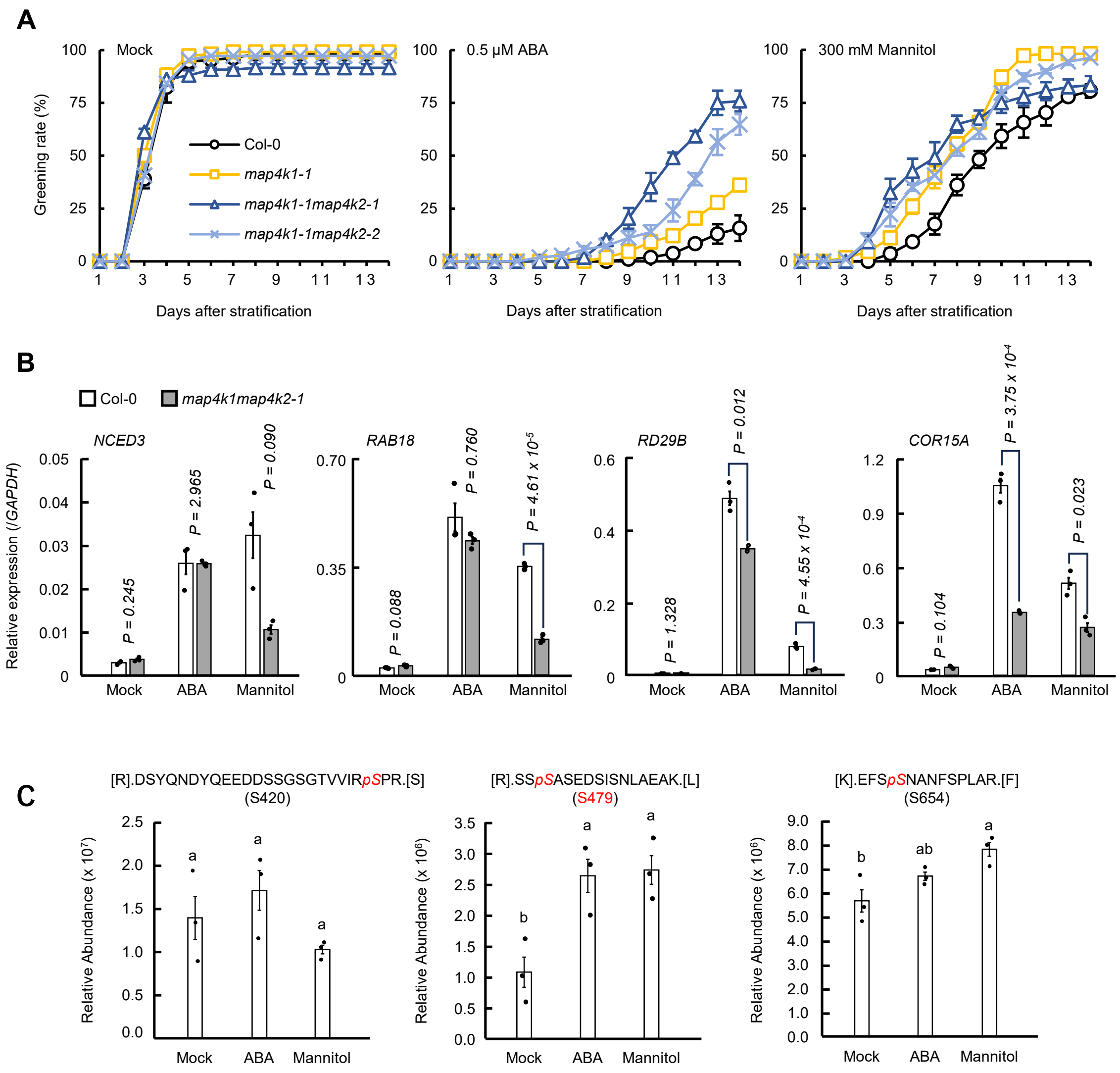

Figure S16. MAP4K1/2 is involved in osmotic stress responses.

A, Cotyledon greening rates of Col-0, *map4k1*, *map4k1map4k2-1* and *map4k1map4k2-2* under conditions of mock, 0.5 µM ABA or 300 mM mannitol treatment. Data presents mean ± standard error (n=3). Each replicate contains 36 seeds. **B**, Relative gene expression levels of *NCED3*, *Rab18*, *RD29B* and *COR15a* in Col-0 and *map4k1map4k2-1* seedlings treated with 50 µM ABA or 400 mM mannitol for 3 h. Bars indicate means ± SE (n = 3), and significance was determined using two-tailed Student's t-test adjusted with Bonferroni. **C**, Quantitative data for phosphopeptides containing Ser-420, Ser-479 or Ser-654 of MAP4K1 were derived from LC-MS/MS analysis of Col-0 seedlings treated with/without 50 µM ABA or 400 mM mannitol for 30 min. Data bars represent means ± SE (n = 3, biologically independent replicate). Different letters indicate significant differences (Tukey's test, $P < 0.05$).

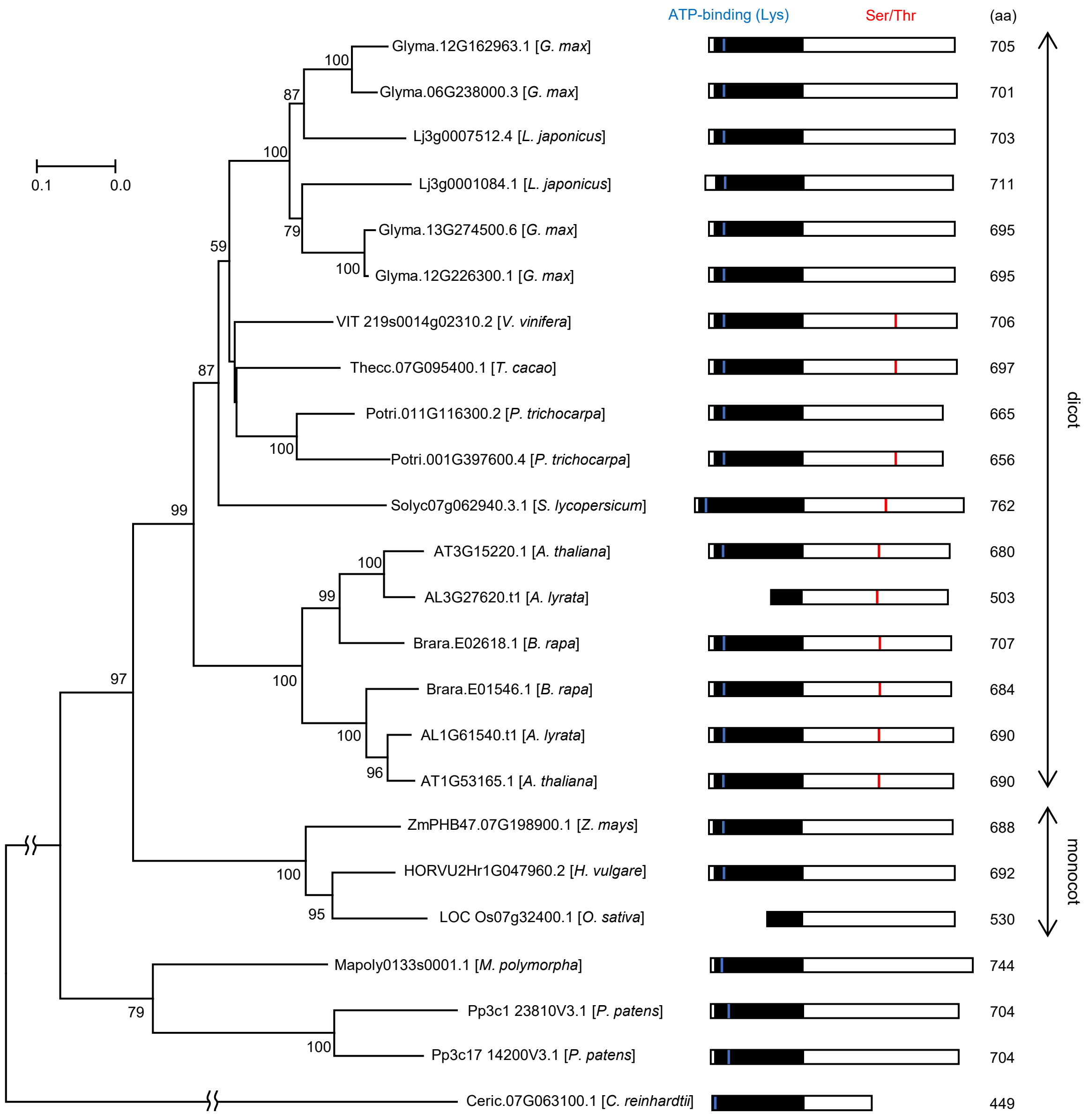

Figure S17. A phylogenetic tree of MAP4K1/2 orthologs in the green lineage.
A schematic of the protein sequences is shown in right, including the position of kinase domain (black). Red line indicates conserved Ser or Thr (Ser/Thr) for *A. thaliana* MAP4K1/2 Ser-479/488. ATP-binding site (Lys) is shown in the bule line. The number in right of each protein shows amino acid length, respectively.

Table S1. All protein IDs described in Fig. S1.

Table S2. Identified protein groups in the proteomic analysis with Arabidopsis guard cells.

Table S3. Identified phosphopeptide groups in the phosphoproteomic analysis with Arabidopsis guard cells.

Table S4. Gene ontology (GO) enrichment analysis in proteomics and phosphoproteomics.

Table S5. IP-MS analysis with GFP or MAP4K1-GFP.

Table S6. Primer sequences used in this study.

Table S7. Scaled abundance of phosphopeptides for heatmap (Fig. 1D)
